# Preserving Metamagnetism in Self-Assembled FeRh Nanomagnets

**DOI:** 10.1021/acsami.2c20107

**Published:** 2023-01-31

**Authors:** Lucie Motyčková, Jon Ander Arregi, Michal Staňo, Stanislav Průša, Klára Částková, Vojtěch Uhlíř

**Affiliations:** † CEITEC BUT, Brno University of Technology, Purkyňova 123, 612 00Brno, Czech Republic; ‡ Institute of Physical Engineering, Brno University of Technology, Technická 2, 616 69Brno, Czech Republic; § Department of Ceramics and Polymers, Brno University of Technology, Technická 2, 616 69Brno, Czech Republic

**Keywords:** self-assembly, FeRh, solid-state dewetting, metamagnetism, antiferromagnetism, supercooling

## Abstract

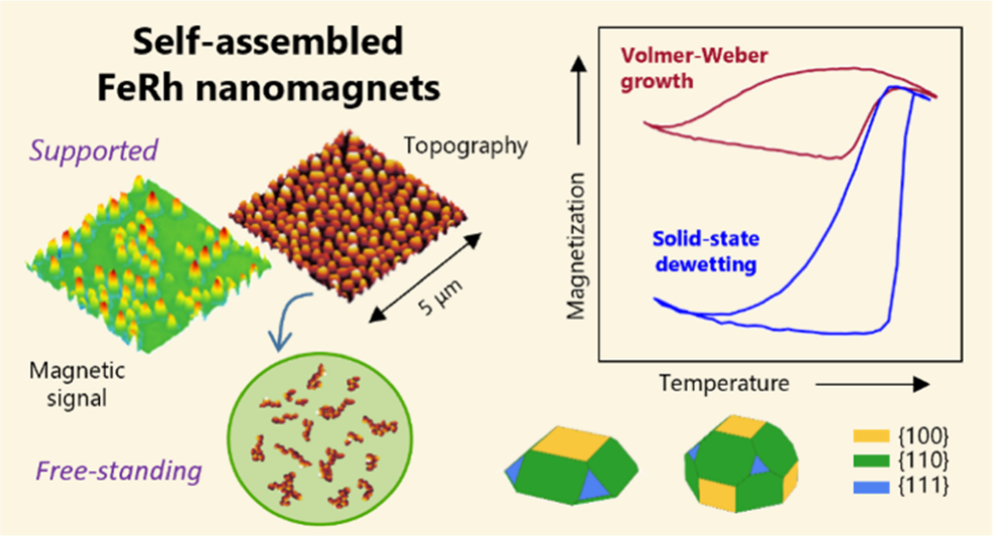

Preparing and exploiting phase-change materials in the nanoscale form is an ongoing challenge for advanced material research. A common lasting obstacle is preserving the desired functionality present in the bulk form. Here, we present self-assembly routes of metamagnetic FeRh nanoislands with tunable sizes and shapes. While the phase transition between antiferromagnetic and ferromagnetic orders is largely suppressed in nanoislands formed on oxide substrates via thermodynamic nucleation, we find that nanomagnet arrays formed through solid-state dewetting keep their metamagnetic character. This behavior is strongly dependent on the resulting crystal faceting of the nanoislands, which is characteristic of each assembly route. Comparing the calculated surface energies for each magnetic phase of the nanoislands reveals that metamagnetism can be suppressed or allowed by specific geometrical configurations of the facets. Furthermore, we find that spatial confinement leads to very pronounced supercooling and the absence of phase separation in the nanoislands. Finally, the supported nanomagnets are chemically etched away from the substrates to inspect the phase transition properties of self-standing nanoparticles. We demonstrate that solid-state dewetting is a feasible and scalable way to obtain supported and free-standing FeRh nanomagnets with preserved metamagnetism.

## 1 Introduction

An ever-present challenge in nanotechnology is the large-scale synthesis of high-quality functional nanostructures with controlled size, shape, and properties (e.g., electronic, optical, magnetic, and chemical).^1^ Self-assembly methods nowadays constitute a particularly efficient tool facilitating high-throughput fabrication of technologically relevant materials, such as catalysts,^2^ fuel cells,^3^ metal–organic frameworks,^4^ and optical metamaterials.^5^ In the domain of magnetic materials, self-assembly also plays a crucial role in the fabrication of superparamagnetic iron-oxide nanoparticles^6^ and granular recording media.^7,8^

While nanostructuring can beneficially lead to emergent phenomena and novel functionalities, it is sometimes desirable that nanomaterials preserve specific bulk properties. In the case of phase-change materials, nanoscale confinement and nanofabrication processing often lead to the unwanted suppression of certain electronic or magnetic ordering states, thus making phase transitions present in the bulk vanish or degrade. For instance, the metal–insulator transition in VO_2_ is mitigated in ultrathin films and nanostructures, where the number of resistivity variation decades is reduced in comparison to the bulk.^9−12^ The deterioration of bulk-like properties upon nanostructuring can be particularly severe in materials with interconnected structural, electronic, and magnetic order parameters. Factors such as excess strain, composition inhomogeneities, grain size effects, or lithographically induced defects can restrain intrinsic material functionalities.^13,14^

Here, we focus on the iron-rhodium (FeRh) alloy, a metallic system featuring a metamagnetic phase transition from the antiferromagnetic (AF) to the ferromagnetic (FM) order above room temperature (*T*_M_ ∼ 360 K).^15^ The phase transition is first-order in nature, only exists in a narrow region near the equiatomic composition range for the CsCl-type structure, and presents a thermal hysteresis of around 10 K.^16^ The sharp magnetization increase upon heating is accompanied by a concomitant isotropic lattice expansion (∼0.5%)^17^ and a reduction in resistivity (∼50%).^18^ Over the last decades, FeRh has been studied as a test-bed for exploring the fundamental physics of coupled order parameters.^19^ The large changes in magnetization, magnetoresistance, and entropy, together with the option to control these changes via various driving forces (e.g., temperature, magnetic field, strain, and light pulses), also make this material interesting for technological applications. FeRh has been proposed for incorporation into magnetic recording^20^ or spintronic^21,22^ devices and is utilized as a model platform for solid-state refrigeration technologies.^23−25^ More recently, FeRh has been considered as a switchable high-contrast label for magnetic resonance imaging with the potential to work near the body temperature.^26,27^

Several fabrication routes have been explored for the self-assembly of nanoscale FeRh elements. On the one hand, solution-phase chemical methods lead to nanoparticles with sizes between 3 and 20 nm.^28,29^ Typically, only a minor fraction of the synthesized sample undergoes the phase transition, which is likely caused by the presence of fcc-ordered FeRh, where the transition is inherently absent.^28,29^ More recently, Cao et al. reported the fabrication of bcc-like particulate FeRh alloys with a more prominent AF–FM phase transition, although the residual magnetization at low temperatures was still relatively large.^30^ Additionally, Biswas et al. have succeeded in obtaining an abrupt phase transition in FeRh powders, which feature interconnected particles of 0.6–1 μm in size.^31^

On the other hand, FeRh nanoislands with sizes in the range of 10–100 nm and supported on crystal substrates have been fabricated using self-organization during physical vapor deposition. This strategy exploits the Volmer–Weber growth mode during high-temperature deposition of FeRh on single-crystal MgO, resulting in the nucleation of physically separated epitaxial islands.^32−34^ Despite the bcc-like structure being favorably imposed via epitaxy, it was concluded that the FM-stabilized surface shell impedes the AF state at the nanoisland core, suppressing the phase transition.^33,34^

These findings underline the exceptionally large sensitivity of metamagnetism in FeRh to different factors, where the phase transition characteristics are affected by stoichiometry, strain and defects,^17,35^ the existence of residual FM-stabilized regions at the interfaces,^36,37^ or nanoscale morphology.^34^ These observations highlight the need for alternative self-assembly routes of phase-change materials to preserve functionalities upon nanoscale size confinement.

In this work, we present the self-assembly of epitaxial sub-micron FeRh nanomagnets with preserved metamagnetism using solid-state dewetting.^38,39^ Starting from thin epitaxial FeRh films on single-crystal substrates, we fabricate arrays of FeRh nanoislands with tunable sizes and shapes. The nanoislands assembled via dewetting sustain the AF–FM phase transition, in contrast to those of comparable size originating from the Volmer–Weber nucleation. We investigate the morphological features of the islands formed upon each assembly route, finding a strong correlation between the existence of metamagnetism and dominant crystal faceting of the nanoislands. This identifies morphology as the leading factor suppressing or allowing the phase transition in FeRh nanoislands. The magnetic phase transition in individual FeRh nanoislands presents extraordinary size-dependent effects, such as extended supercooling (>150 K). Finally, the nanoislands are chemically etched from the substrates to obtain metamagnetic FeRh nanoparticles in solution.

## 2 Results and Discussion

### 2.1 Self-Assembly of FeRh Nanoislands

We report two distinct processes that combine magnetron sputter deposition and annealing, leading to the self-assembly of sub-micron FeRh islands on single-crystal MgO(001), MgO(011), and Al_2_O_3_(0001) substrates. Both routes are triggered by thermodynamic surface energy minimization and driven by high-temperature annealing during and/or after the growth. For the sake of simplicity, we initially describe the MgO(001) substrate system.

**Figure 1:**
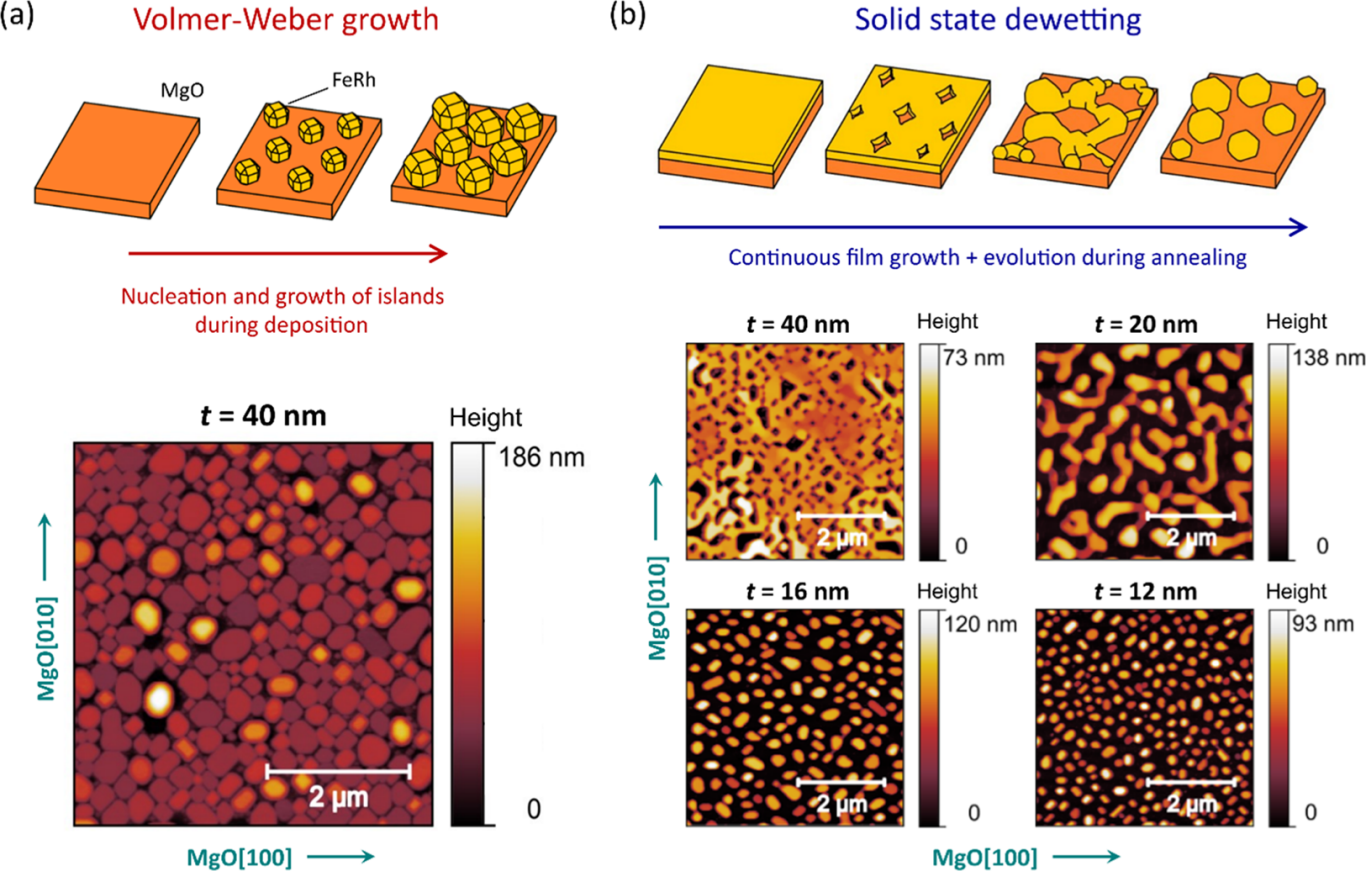
(a) Schematic representation of the nanoisland self-assembly process via Volmer–Weber nucleation (top panel) and the exemplary topographic height profile dataset for a sample with a nominal *t* = 40 nm thickness (bottom panel). (b) Illustration of the solid-state dewetting process in FeRh thin films (top panel), and topographic height profile datasets for FeRh deposits formed via solid-state dewetting for different film thicknesses *t* (bottom panel). As *t* is reduced, the film morphology evolves from a continuous coverage with square-shaped voids toward well-separated, sub-micron islands of decreasing size.

In the first process, FeRh growth is initiated at 750 K with a deposition rate of 2 nm min^–1^. After 3 min of deposition, the substrate temperature is ramped within 5 min to 1100 K, maintaining this temperature until the film is completed with a nominal thickness *t*. The sample is subsequently annealed at 1100 K for 80 min. This process does not lead to the formation of a continuous FeRh film but rather results in the nucleation of separated islands on the MgO substrate (Figure 1a, top panel). The surface morphology of an FeRh sample fabricated via this procedure for *t* = 40 nm is characterized via atomic force microscopy (AFM) and shown in Figure 1a (bottom panel). The deposit consists of densely packed sub-micron islands extending over the entire substrate. The islands display a characteristic rectangular shape with pronounced faceting along the [100] and [010] axes of FeRh (in Figure 1a, these axes are 45° rotated with respect to the image edges, corresponding to the principal axes of MgO). X-ray diffraction (XRD) measurements confirm the attainment of the CsCl-type structure and a well-defined FeRh(001) crystallographic texture (Figure S1, Supporting Information).

The formation of FeRh islands originates from surface energy optimization leading to a preferential Volmer–Weber growth mode. We find that film percolation does not occur in the first 3 min of the deposition (at a nominal film thickness of 6 nm). Ramping the growth temperature to 1100 K before forming a continuous layer results in Volmer–Weber growth due to the large surface energy difference between the deposit (γ_FeRh,100_ = 2.17 J m^–2^)^34^ and the substrate (γ_MgO,100_ = 1.15 J m^–2^).^40^ Nanoisland size analysis reveals a bimodal distribution with a broad peak at the equivalent diameter value of 260 nm and the additional presence of sub-50 nm islands (Figure S1, Supporting Information). Scanning electron microscopy (SEM) observations reveal the ubiquitous presence of these smaller nanoislands intercalated between bigger ones (>100 nm), further confirming the scenario of Volmer–Weber nucleation (Figure S2, Supporting Information).

This result is in line with previous reports of island-like growth in high-temperature-deposited (>900 K) ultrathin FeRh/MgO(001) samples.^32−34^ Deposition of an FeRh film at constant, elevated temperatures above 1000 K also led to the nucleation of micron-sized islands of arbitrary shapes on MgO(001) (Figure S3, Supporting Information), which we attribute to a complex balance between the temperature-dependent surface energy, deposit-to-substrate mismatch, and strain relaxation during growth and post-growth annealing.

The second self-assembly process, developed in this work, is initiated by the non-equilibrium growth of a continuous metastable FeRh thin film on MgO. FeRh is sputtered at a substrate temperature of 750 K throughout the entire deposition. The samples are subsequently annealed at 1100 K for 80 min. We find that the thermal load exerted during post-growth annealing is enough to drive metastable FeRh films toward the thermodynamically favored island-like morphology via solid-state dewetting.^38,39^

Spontaneous agglomeration of three-dimensional islands starts with the nucleation and deepening of grooves in the epitaxial FeRh film (Figure 1b, top panel), a process that initiates at defect sites consisting of vacancies or contaminants.^38^ This step is followed by the anisotropic retraction and thickening of faceted rims around the voids, leading to hole nucleation down to the substrate.^41,42^ The occurrence of further mass transport in the form of capillary instabilities and perturbations in front of the receding rims, finger formation, and Rayleigh-type instabilities^43^ leads to the self-assembly of sub-micron FeRh islands (Figure 1b).

AFM images of dewetted FeRh films are shown in the bottom panel of Figure 1b. The resulting morphology is strongly dependent on the nominal film thickness. For *t* = 40 nm, FeRh/MgO samples feature an interrupted film morphology with square-shaped grooves that point toward a strongly faceted void growth along the [100] and [010] crystal axes of FeRh. Lowering *t* leads to more advanced dewetting scenarios, in accordance with the inverse dependence of the rim retraction rate with film thickness.^38,39^ The morphology of the deposit (Figure 1b, bottom panel) evolves from maze-like, interconnected islands (*t* = 20 nm) toward physically well-separated sub-500 nm nanoislands (*t* = 12, 16 nm). We have monitored in situ the onset of void nucleation and growth during annealing by measuring low energy ion scattering (LEIS) on pre-grown FeRh continuous films of different thicknesses. The emergence of voids, marked by the appearance of a scattering signal from the Mg and O substrate atoms at the surface, is triggered for temperatures values of ∼800–850 K, with their growth steadily occurring up to 1100 K (Figure S4, Supporting Information). Dewetted FeRh nanoislands form arrays over the whole substrate area, with the crystallographic FeRh(001) out-of-plane texture persisting after solid-state dewetting (Figure S1, Supporting Information).

Well-separated nanoislands (*t* = 12, 16 nm) feature a characteristic oval shape with slight elongations along the principal crystal axes of FeRh, originating from the anisotropic void growth and rim faceting during dewetting. Compared to the Volmer–Weber nucleated ones, dewetted nanoislands apparently display a larger number of crystal facets, giving them a rounder geometric appearance (Figures 1b and S2, Supporting Information). Furthermore, contrary to the Volmer–Weber growth mode, there is no presence of smaller intercalated nanoislands between the larger ones. Decreasing the deposited nominal thickness allows achieving nanoisland sizes down to the ∼100 nm range (Figures 1b and S1, Supporting Information).

As a general observation, we found that the FeRh film percolation is compromised around the nominal thickness of ∼10 nm and below, identifying a cross-over between solid-state dewetting and Volmer–Weber nucleation. The exact threshold strongly depends on the sample-to-sample growth temperature variations caused by the substrate-to-holder thermal contact. Hence, the lowest achievable thickness of the continuous film constitutes an intrinsic limit for decreasing the size of nanoislands formed via solid-state dewetting. Besides, the resulting size of the dewetted nanoislands depends on the density of groove nucleation sites in the initial stage of dewetting (Figure S5, Supporting Information). Groove nucleation can be triggered by pinholes in the film, impurities, dislocations, and topographical irregularities on the substrate (e.g., terraces^38^). A larger density of hole nucleation sites will typically break up the film into a larger amount of smaller nanoislands.

The detailed morphology and crystal faceting of self-assembled nanoislands were additionally analyzed on MgO(011) and Al_2_O_3_(0001), where we also obtained FeRh nanoisland arrays via dewetting. Dewetting of FeRh on Al_2_O_3_(0001) is also facilitated by the low surface energy of this surface plane with γ_Al_2_O_3_,0001_ = 1.4 J m^–2^.^44^ MgO(011) possesses a higher surface energy of γ_MgO,110_ ∼ 3 J m^–2^,^45^ making island assembly thermodynamically unfavorable. However, moderate micro-faceting of the substrate surface lowers this value to about ∼1.7 J m^–2^,^46^ hence explaining the occurrence of dewetting in our experiments.

**Figure 2:**
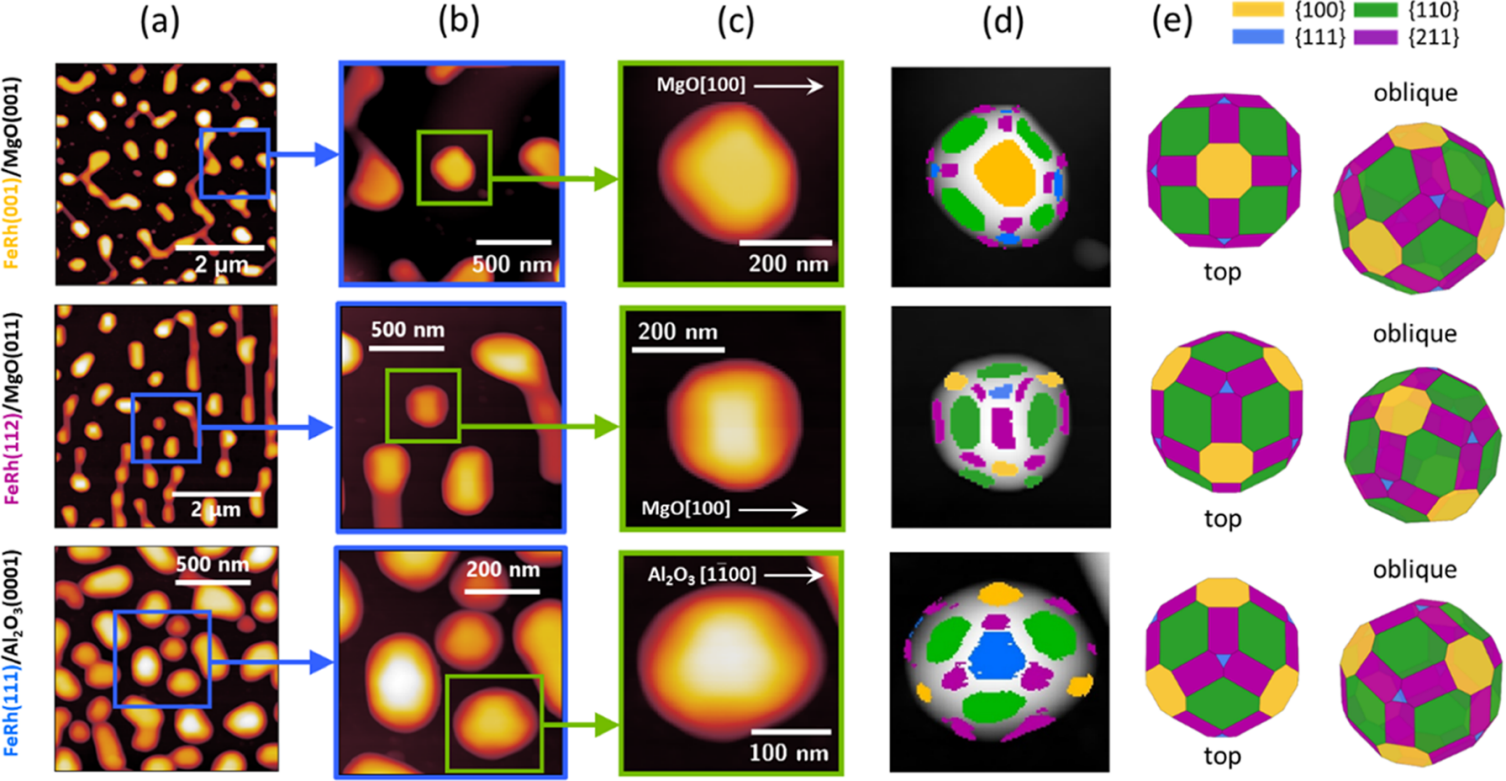
Morphology and faceting of self-assembled epitaxial FeRh nanoislands on single-crystal oxide substrates. (a) Topographic AFM data with tens of nanoislands for samples with FeRh(001), FeRh(112), and FeRh(111) out-of-plane crystallographic orientations (*t* = 16 nm). (b,c) shows zoomed-in topographic scans showing a few nanoislands and a single nanoisland, respectively. (d) Facet analysis obtained from the high-resolution, single-nanoisland topography scan. (e) Top and oblique views of the Wulff construction for each of the different nanoisland textures.

Figure 2a shows an overview of the AFM scans of FeRh nanoislands obtained via solid-state dewetting on MgO(001), MgO(011), and Al_2_O_3_(0001). The nanoislands possess a high crystalline order with (001), (112), and (111) out-of-plane textures, respectively (Figures S6 and S7, Supporting Information). The FeRh(001)/MgO(001) and FeRh(111)/Al_2_O_3_(0001) epitaxial matchings have already been described for continuous films,^16,17^ whereas we find that the epitaxy of FeRh on MgO(011) follows a pattern common for elemental bcc metals like Fe and Cr, showing (112)-oriented preferential growth on MgO(011).^47,48^

Anisotropic rim retraction following void formation during solid-state dewetting strongly determines the shape of self-assembled nanoislands. Topographic characterization of FeRh films during early dewetting stages (Figure S8, Supporting Information) hints to nanoisland arrangements with characteristic fourfold, twofold, and sixfold symmetries for the (001), (112), and (111) oriented cases, respectively. FeRh(001) nanoislands elongate along the [100] and [010] axes of FeRh (Figure 2a). Furthermore, FeRh(112) nanoislands predominantly elongate along the FeRh[111]||MgO[011] direction (vertical direction in Figure 2a), sometimes forming high aspect-ratio needles joining the nanoislands. Finally, FeRh(111) nanoislands typically exhibit more circular shapes, with a few instances of elongated oval islands. For the latter two substrates, we observe more densely packed and smaller nanoislands than those obtained on MgO(001), as a larger density of hole nucleation sites arises from the higher FeRh-to-substrate epitaxial mismatch and the subsequent larger presence of stacking faults and dislocations (Figure S9, Supporting Information).

The shape of selected nanoislands ∼200 nm in size has been studied in detail for the distinct out-of-plane crystallographic orientations (zoomed-in AFM images in Figure 2b,c). The analysis of surface normal orientation distributions in high-resolution topographic scans allows crystallographic facet identification (see Experimental Section). Figure 2d shows the marked crystal facets superimposed with topography, side-by-side to the bare data in Figure 2c, upon considering the {100}, {110}, {111}, and {211} crystal facets of FeRh.

We have modeled the equilibrium crystal shapes or Wulff constructions (see Experimental Section) of FeRh crystals using the surface energies calculated by Liu et al.,^34^ with γ_100_ = 2.17 J m^–2^, γ_110_ = 2.10 J m^–2^, and γ_111_ = 2.37 J m^–2^. In addition, we set γ_211_ = 2.20 J m^–2^ to match the topography line profiles to the equilibrium crystal shapes (Figure S10, Supporting Information). The modeled shapes for each nanoisland texture are shown in Figure 2e. The experimentally determined nanoisland faceting and equilibrium shapes (Figure 2d,e) agree very well, indicating that dewetting leads to the assembly of equilibrium FeRh crystal shapes. The facet analysis also confirms the epitaxial matching relations deduced from XRD (Figure S7, Supporting Information).

### 2.2 Magnetic Properties of FeRh Nanoislands

**Figure 3:**
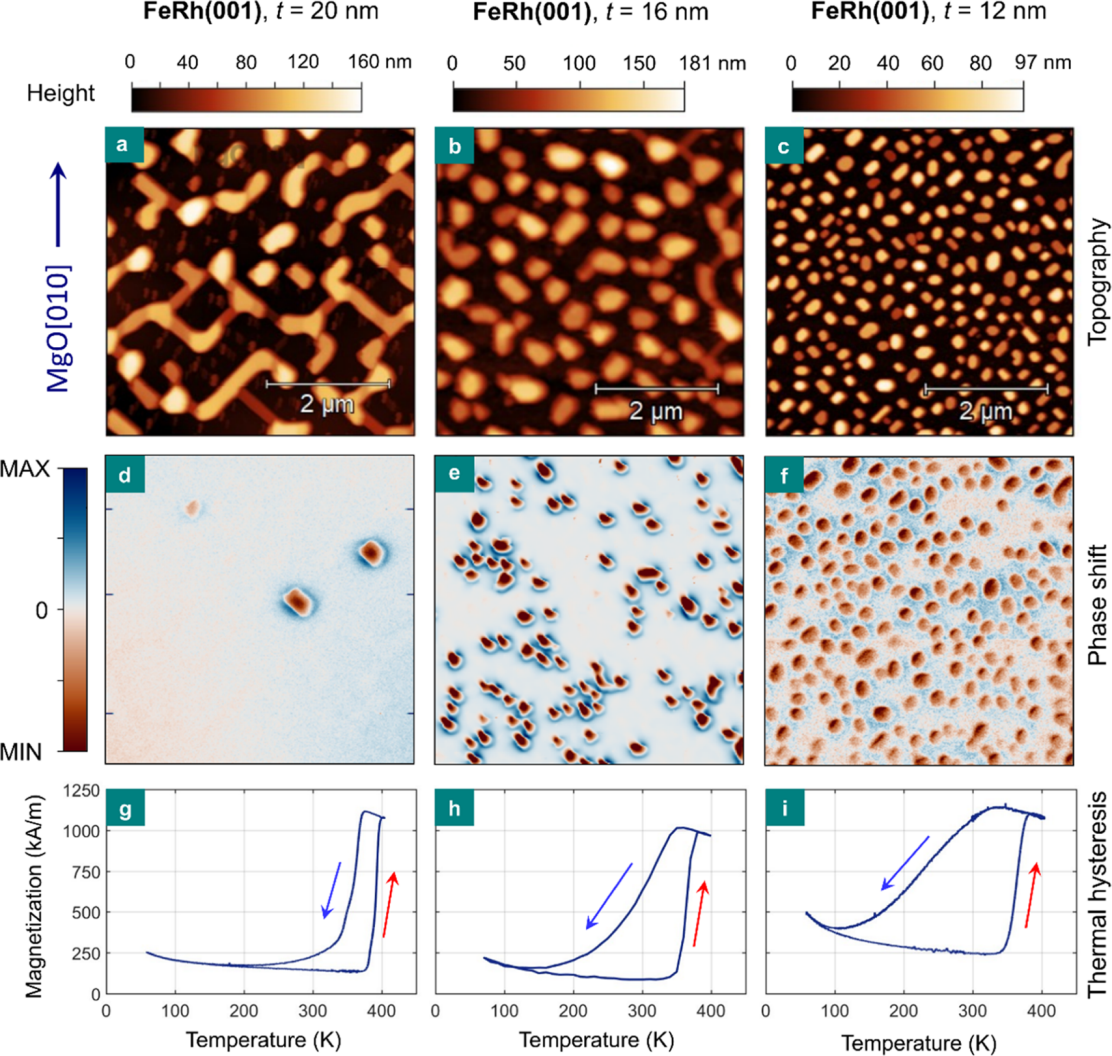
Room-temperature magnetic properties and phase transition in FeRh nanoislands. (a–c) AFM topography images over a 5 × 5 μm^2^ area of nanoisland samples with *t* = 20, 16, and 12 nm. (d–f) Room-temperature MFM measurements over the same sample area. The inset in (a) indicates the crystallographic in-plane direction of the micrograph, which is also valid for all panels (b–f). (g–i) Temperature dependence of the magnetization in the range of 55–400 K for the samples described above. The arrows indicate the heating and cooling cycles in the thermal hysteresis.

The magnetic properties of the FeRh nanoislands assembled via solid-state dewetting are first analyzed at room temperature. Figure 3a–c show AFM micrographs of a 5 × 5 μm^2^ area containing sub-micron nanoislands with (001) out-of-plane texture and different nominal thicknesses. The room-temperature magnetic force microscopy (MFM) measurements in Figure 3d–f correspond to the topographic scans displayed above. The magnetic signal principally arises from out-of-plane oriented magnetic moments due to the externally applied vertical magnetic field (see Experimental Section). The morphology of islands with *t* = 20 nm (Figure 3a) corresponds to a maze-like structure with well-recognizable dewetted features. The magnetic signal (Figure 3d) is only present in three distinct regions, which disrupt the otherwise prevailing zero contrast background (represented by the white color in Figure 3d). The major fraction of FeRh islands thus manifests zero magnetic signal at room temperature.

Nanoislands corresponding to *t* = 16 nm show similar features compared to the sample with *t* = 20 nm, but the fraction of islands showing magnetic contrast is larger (Figure 3b,e). About half of the well-separated islands exhibit zero MFM signal, with the remaining half revealing a clear FM ordering (Figure 3e). The relative population of islands showing a magnetic signal is even larger for the sample with *t* = 12 nm, containing well-separated ∼200 nm nanoislands (Figure 3c,f), where only a few islands show zero magnetic signal. This island-size-dependent analysis thus reveals that with decreasing size, a larger fraction of nanoislands displays a clear FM ordering at room temperature. The magnetic properties of FeRh nanoislands with (112) and (111) textures were also evaluated. MFM measurements indicate that almost all FeRh(112) nanoislands remain FM at room temperature. In the case of FeRh(111), about half of the ∼100 nm nanoislands show a significant MFM signal (Figure S9, Supporting Information).

In order to characterize the phase transition in the nanoisland samples, the temperature dependence of magnetization was measured using vibrating sample magnetometry (VSM). The magnetization data are shown for each sample in the panels below the corresponding topographic and MFM data (Figure 3g–i), where all FeRh(001) nanoisland samples undergo a prominent phase transition. Overall, the heating cycle of the thermal hysteresis shows an abrupt phase transition, while the magnetization drop during the cooling cycle is more gradual upon decreasing the size of the nanoislands. For larger nanoislands, the phase transition during cooling is abrupt and only shows a slight tail, marking the need for a cool-down slightly below room temperature in order to complete the transition (Figure 3g).

As the nanoislands’ size decreases, the thermal hysteresis features a more gradual change of magnetization during cooling (Figure 3h,i). For these small nanoislands, a considerable fraction of high-temperature magnetization is retained at room temperature during cooling, and the phase transition is only completed after cooling down the sample below 150 or 100 K. This observation agrees well with the large fraction of nanoislands showing a magnetic MFM signal at room temperature (Figure 3e,f). We conclude that a large fraction of nanoislands with sizes around and below 200 nm remain supercooled in the FM phase at room temperature. This behavior is also found in the case of (112) and (111)-textured nanoislands, where a prominent phase transition is equally present (Figure S9, Supporting Information).

**Figure 4:**
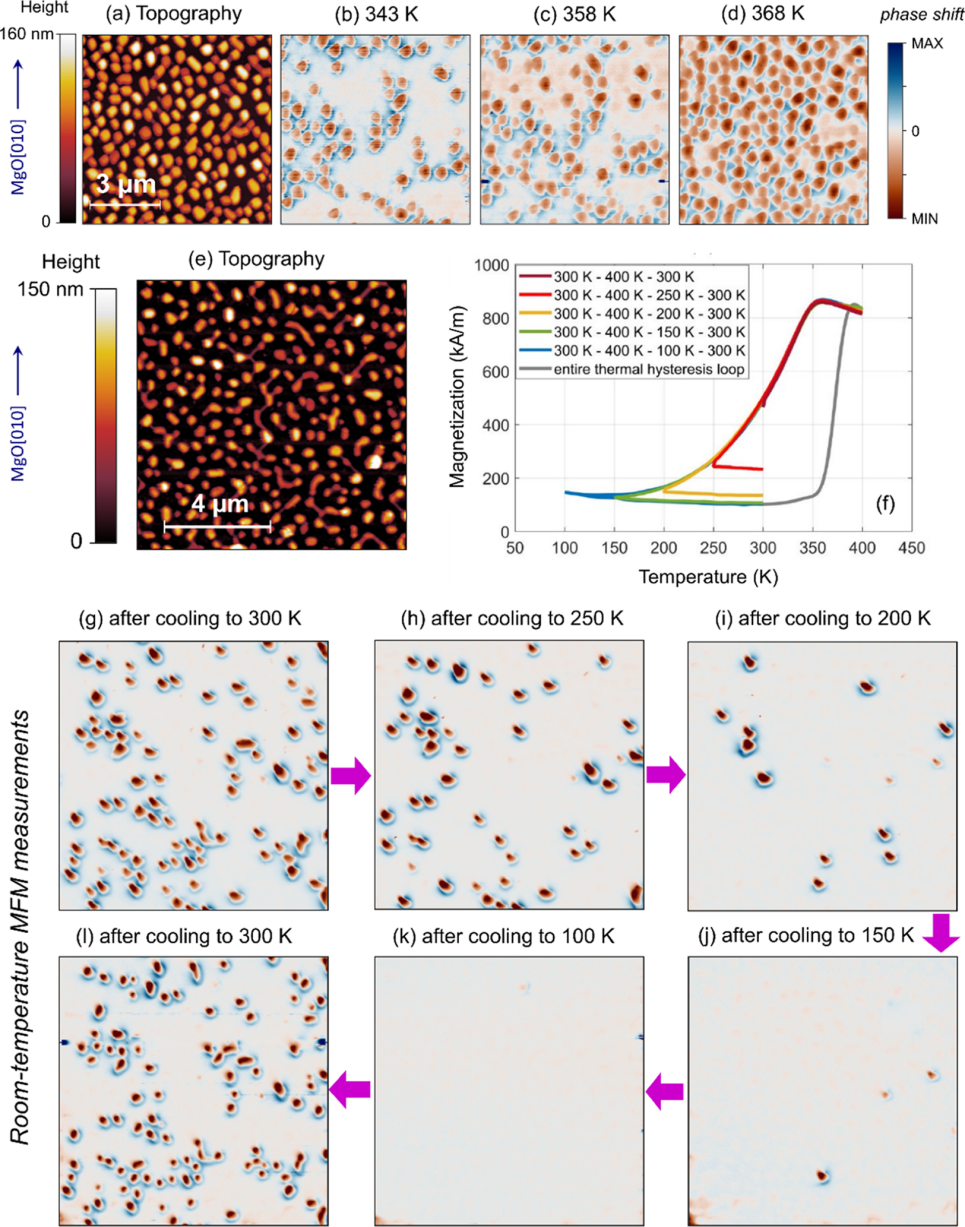
Phase transition and supercooling in FeRh nanoislands with *t* = 16 nm. (a) AFM image and (b–d) MFM images of FeRh(001) nanoislands over an 8 × 8 μm^2^ area during heating. (e) AFM image of over a 10 × 10 μm^2^ area. (f) Magnetization vs temperature for the sequential thermal cycling employed before room temperature MFM characterization. (g–l) Room-temperature MFM measurements upon warming up the sample to 400 K and subsequently cooling down to the temperatures indicated in each panel. The arrows between panels indicate their sequential order.

In the following, we present the magnetic behavior of individual dewetted nanoislands across the phase transition. Figure 4a shows the topography of FeRh(001) islands (*t* = 16 nm) over an 8 × 8 μm^2^ area. As the nanoislands are first heated across the AF-to-FM phase transition, most of them become FM within the temperature range of 343 to 368 K, as evidenced by a clear magnetic signal in the MFM scan (Figure 4b–d).

The cooling characteristics are investigated over a larger 10 × 10 μm^2^ sample area (Figure 4e). Here, we combine ex situ cooling (down to 100 K) and heating (up to 400 K) of the samples with posterior MFM observation at room temperature. Figure 4f shows temperature-dependent magnetization data for the different heating/cooling protocols performed prior to the room-temperature MFM characterization. The initial magnetic configuration of the islands at room temperature is shown in Figure 4g. Apparently, a certain fraction of the islands is in the FM phase at room temperature. The sample is subsequently warmed up to 400 K, followed by a cool-down to 250 K. Imaging the room-temperature magnetic configuration after this protocol reveals that a number of islands that were in the FM phase before show no magnetic signal after the additional cool-down (Figure 4h), suggesting that they were supercooled at room temperature and underwent the FM-to-AF phase transition upon additional cooling.

The temperature protocol and imaging are repeated upon first warming up the sample to 400 K in each step and subsequently cooling the sample to 200 K (Figure 4i), 150 K (Figure 4j), and 100 K (Figure 4k). The number of FeRh islands in the FM phase decreases upon each consecutive cooling protocol. After cooling down to 150 K, only three nanoislands remain FM (Figure 4j), and finally, we find that all nanoislands have transitioned to the AF phase upon cooling down to 100 K (Figure 4k).

The supercooled nanoislands transition to the FM phase well above 300 K, in the range ∼350–370 K, regardless of the thermal cycling protocol. This indicates that sub-micron FeRh nanoislands can present very extensive supercooling at about 150–200 K below their transition temperature (for comparison, the deep supercooling regime for the liquid-to-ice phase transition in water is ∼43 K below the freezing point^49^). While supercooling of about ∼10–20 K has been previously reported in lithographically patterned FeRh wires,^50,51^ we observe that self-assembled FeRh nanoislands are capable of sustaining much deeper supercooled FM states. Finally, the sample is warmed up to 400 K and cooled down to 300 K to obtain an additional snapshot of its magnetic state (Figure 4l). The magnetic order of the nanoislands closely resembles that of the initial state at 300 K (Figure 4g), but a few additional islands seem to be in the FM state.

These findings altogether point to the extraordinary sensitivity of the phase transition in confined FeRh structures to factors such as defects, availability of nucleation sites, and thermal activation. In particular, the decrease in the number of AF phase nucleation sites upon reducing the nanomagnet size seems to be behind the observation of the very pronounced supercooling. This aspect could also be at the origin of the complete suppression of the phase transition in FeRh at the ∼10 nm scale and below, as observed in highly ordered nanoparticles embedded in a carbon matrix, where the FM phase persists down to 2 K.^52,53^

Another interesting observation is the complete suppression of phase separation in FeRh nanoislands across the phase transition. We did not observe any coexistence of AF and FM domains upon temperature cycling, indicating that the abrupt nature of the first-order phase transition is recovered within each nanoisland.

The emergence of asymmetric thermal magnetization hysteresis, with a relatively abrupt transition during heating and a broad transition during cooling, has been frequently observed in FeRh specimens in the literature. Examples include fine particle and powder systems synthesized via solid-phase reduction and mechanochemical methods.^30,31^ A particularly prominent case is that of (ultra)thin FeRh films grown on single-crystal oxide substrates, which often turn out to be discontinuous or granular.^54−58^ We suggest that a substantial presence of supercooled nanoscale grains within FeRh films could explain the appearance of such asymmetric thermal hysteresis. It is worth noting that engineering the sputter process can improve to a certain degree the continuity and smoothness of ultrathin FeRh films on oxide substrates.^59^

### 2.3 Morphology-Enabled Phase Transition

**Figure 5:**
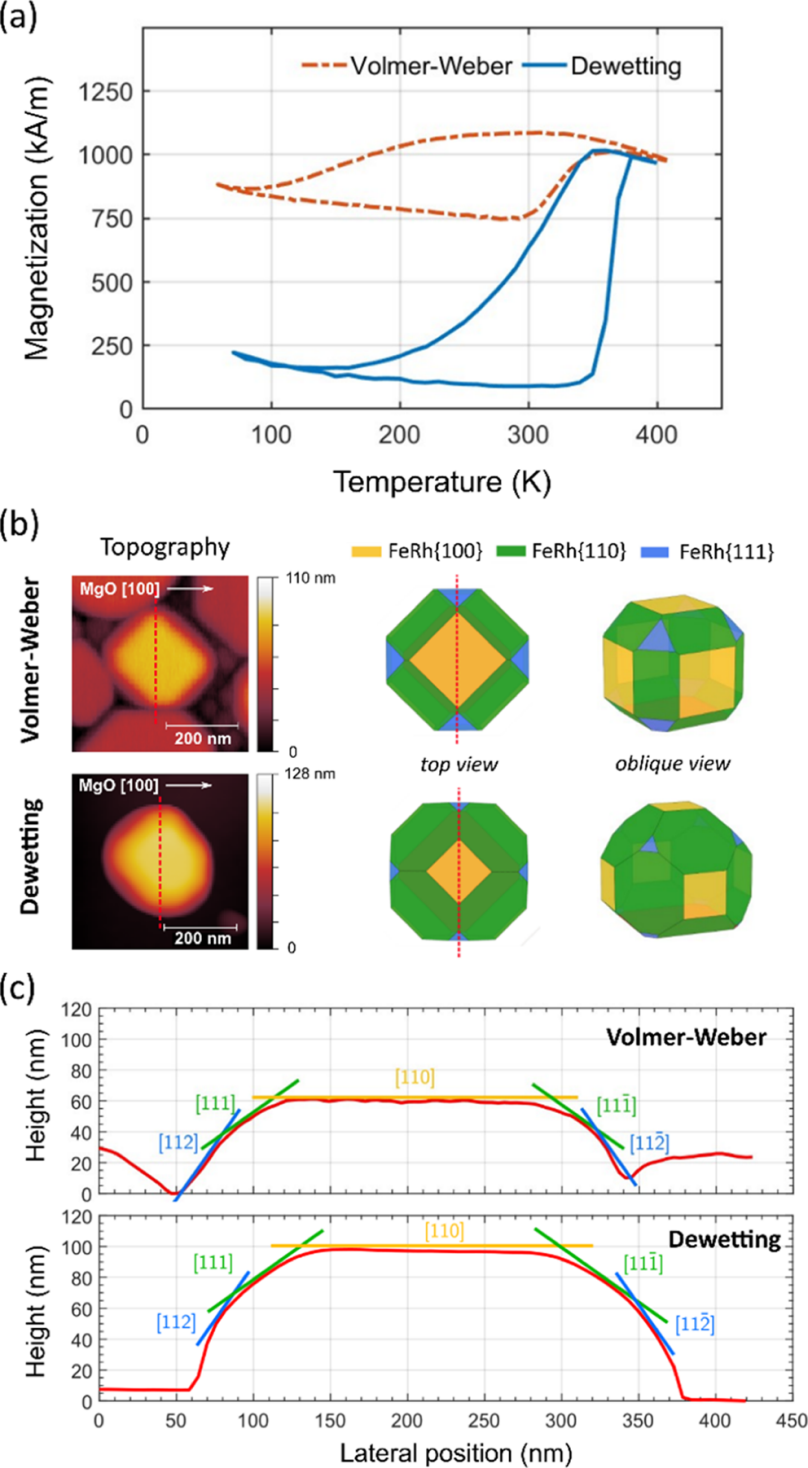
Morphology and metamagnetism in FeRh nanoislands. (a) Magnetization vs temperature measurements for FeRh(001)/MgO(001) nanoislands assembled via Volmer–Weber growth (*t* = 40 nm) and solid-state dewetting (*t* = 16 nm). (b) Topography scans showing a single nanoisland of ∼300 nm in diameter for both Volmer–Weber growth and solid-state dewetting. Wulff constructions of FeRh nanocrystals obtained upon considering the surface energies of the FM and AF phases are shown side-by-side to the topography scans, which resemble Volmer–Weber-nucleated and dewetted nanoisland morphologies, respectively. The representation of {211} planes has been omitted for clarity. (c) Topography line scans of Volmer–Weber and dewetted single nanoislands extracted from the data in (b). The indices within brackets in (c) denote the crystallographic directions along the line scan for each facet.

While the metamagnetic behavior is preserved in dewetted FeRh nanoislands with lateral sizes of ∼300 nm and below, the phase transition is strongly suppressed in Volmer–Weber nucleated nanoislands of similar or larger size (Figure 5a). First-principles calculations by Liu et al.^34^ point to a strong link between a given magnetic phase and the surface energy of the principal FeRh crystal facets. The minimum surface energy, and thus the preferential faceting orientation, is predicted for the {110} planes in the AF phase, whereas the {100} planes are the ones with the lowest surface energy in the FM phase (see Table 1).

**Table 1:** Surface Energy Values for the Main Facets of FeRh^a^

FeRh plane	γ[J m^–2^] AF phase	γ[J m^–2^] FM phase	reference
{100}	2.17	1.78	(34)
{110}	2.10	1.88	(34)
{111}	2.37	2.08	(34)
{211}	2.20		this work

a The values from Liu et al. are calculated by density functional theory for both AF and FM bulk phases;^34^ we assume a Rh-terminated surface for each case and FM surface configurations in the AF phase.^36,37^ For {110} planes, both Fe and Rh atoms are present at the surface. The energy for the {211} planes is obtained by matching the experimental topographic profiles (Figure S10, Supporting Information).

Figure 5b shows AFM scans for selected nanoislands in the Volmer–Weber-nucleated and dewetted samples, respectively. Both nanoislands are similar in lateral size, but their morphology is qualitatively different. Apparently, the island assembled via Volmer–Weber nucleation shows a prevailing {100} crystal faceting with a characteristic rectangular shape, while the dewetted island has a rounder morphology arising from the predominant {110} crystal plane faceting. Topographic line scans along the FeRh[110] direction (Figure 5c) reveal that both nanoislands correspond to nanocrystals truncated above their centers. However, while the Volmer—Weber-nucleated island features a relatively low height-to-diameter ratio and a prominent faceting for the {100} planes, the dewetted island has a noticeably larger height in proportion, with a predominant presence of the {110} facets.

It is interesting to notice that the Wulff nanocrystal models obtained by choosing the surface energy values for the AF or FM phases (Table 1) qualitatively predict the experimental nanoisland shapes (Figure 5b). That is, equilibrium FM FeRh nanocrystals resemble the morphology of FeRh nanoislands obtained via Volmer–Weber nucleation, while AF FeRh nanocrystals resemble nanoislands assembled via solid-state dewetting (Figure 5b).

**Figure 6:**
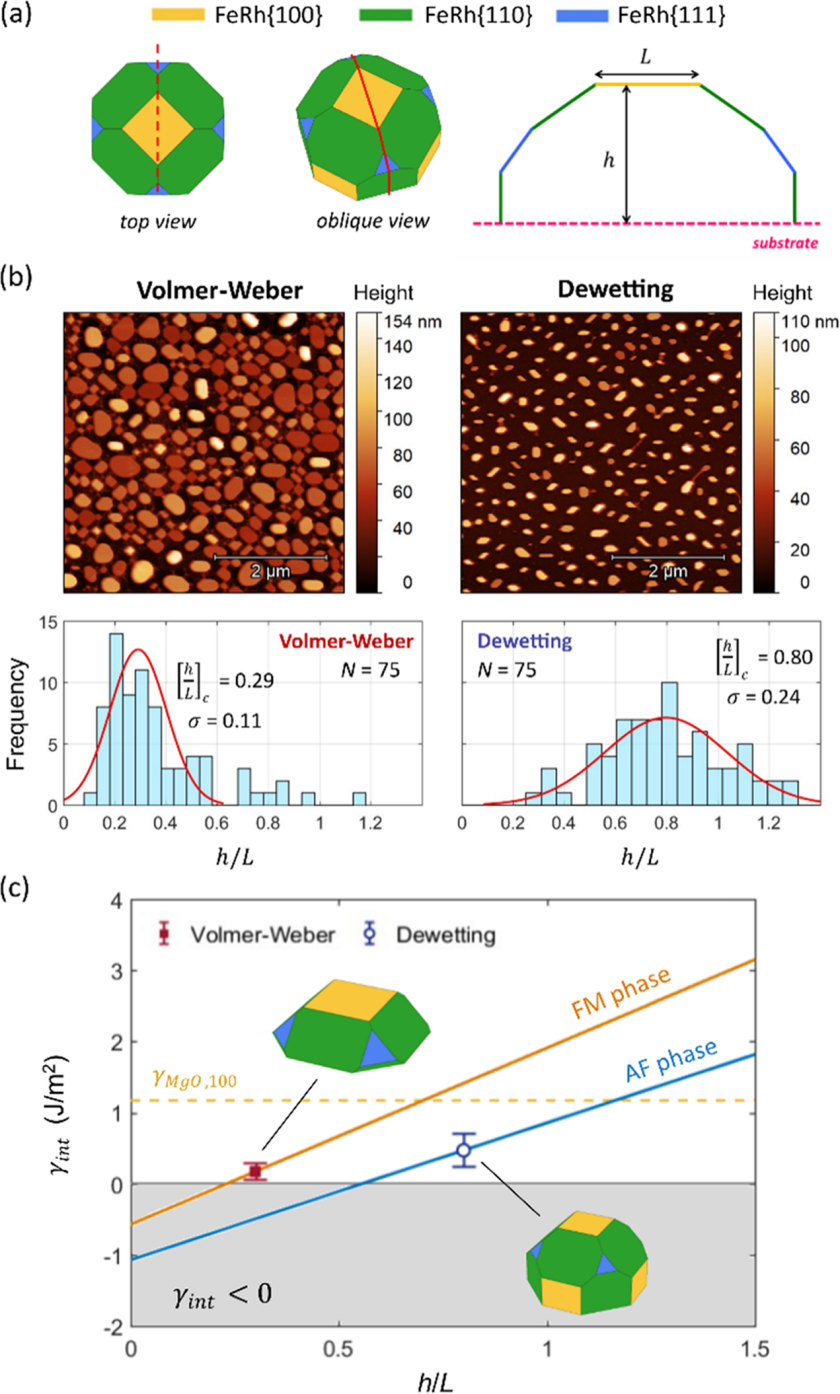
Shape analysis of FeRh nanoislands. (a) Top and oblique views of the Winterbottom construction for a (100)-oriented nanoisland truncated above the nanocrystal center. The {211} planes are omitted for clarity. The red dashed lines indicate the line-scan orientation measured for each island. On the right, schematics of the nanoisland attributes (height *h*, cusp width *L*) extracted from the line scans. (b) High-resolution AFM image (5 × 5 μm^2^) and histograms of the measured *h*/*L* ratio for *N* = 75 nanoislands obtained via Volmer–Weber nucleation and dewetting. The fitted central *h*/*L* value and the standard deviation are indicated (for Volmer–Weber nanoislands, outliers with *h*/*L* > 0.6 are neglected). (c) FeRh/MgO interface energy vs *h*/*L* considering the AF and FM phases of FeRh (solid lines), and values obtained from the measured *h*/*L* distributions for Volmer–Weber-nucleated and dewetted nanoislands. The schematics in the inset in (c) show the shapes of the truncated nanocrystals in each case.

To further elucidate the contrasting magnetic behavior of nanoislands assembled via different routes, we have thoroughly analyzed the topographic features of nanoisland ensembles on MgO(001) substrates formed via Volmer–Weber growth and solid-state dewetting. Two characteristic length scales are measured from each island or truncated crystal: the base to cusp height *h* and the extent of the cusp *L* in the FeRh[110] direction (see Figure 6a). We perform a statistical analysis of the *h*/*L* ratio by considering 75 islands from each sample (Figure 6b), confirming the morphological differences anticipated in Figure 5c for the two types of nanoislands. The central value obtained from the *h*/*L* histogram is 0.3 ± 0.1 for Volmer–Weber-nucleated islands and 0.8 ± 0.2 for those assembled via solid-state dewetting, thus highlighting a marked difference in the resulting shape of the nanoislands assembled via each route.

Next, we employ Wulff–Kaischev’s theorem, which mathematically relates the occurrence and geometry of the facets in a supported crystal with the surface energy values of the crystal and the substrate, as well as with the interface formation energy.^60,61^ Following this approach, we arrive at the following expression (Note S1, Supporting Information)
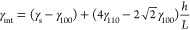
1which relates the FeRh/MgO interface energy to the measured nanoisland *h*/*L* ratio and surface energies of FeRh and the MgO substrate. Figure 6c shows the dependence of the interface energy on *h*/*L* according to eq 1 upon considering AF or FM surface energy values. Negative γ_int_ values do not represent an accessible physical solution, and values with γ_int_ > γ_s_ ≈ 1.17 J m^–2^ correspond to nanocrystals truncated below their geometric center, which were not experimentally observed.

Using the central *h*/*L* values in the histogram for the two types of islands, we observe that for a morphology corresponding to that of Volmer–Weber-nucleated islands, the only allowed interface energy exists upon assuming FeRh surface energy values in the FM phase (*h*/*L* = 0.3, γ_int_ = 0.17 J m^–2^), while for dewetted nanoislands, both phases are accessible, with the AF phase representing the more stable configuration (*h*/*L* = 0.8, γ_int_ = 0.47 J m^–2^) and the only one corresponding to a truncation above the nanocrystal center.

The manifested differences in the shape and magnetic phase transition properties of the FeRh nanoislands formed via different assembly routes point to a very strong connection between their nanocrystal morphology and the favored magnetic order. Our study provides strong evidence for this connection, and we can conclude that FeRh nanoislands with distinctive shapes tend to sustain or preclude the AF phase, and in turn, metamagnetism. This scenario is compatible with the phase-dependent calculated surface energies of FeRh.^34^

### 2.4 Free-Standing FeRh Nanoparticles

**Figure 7:**
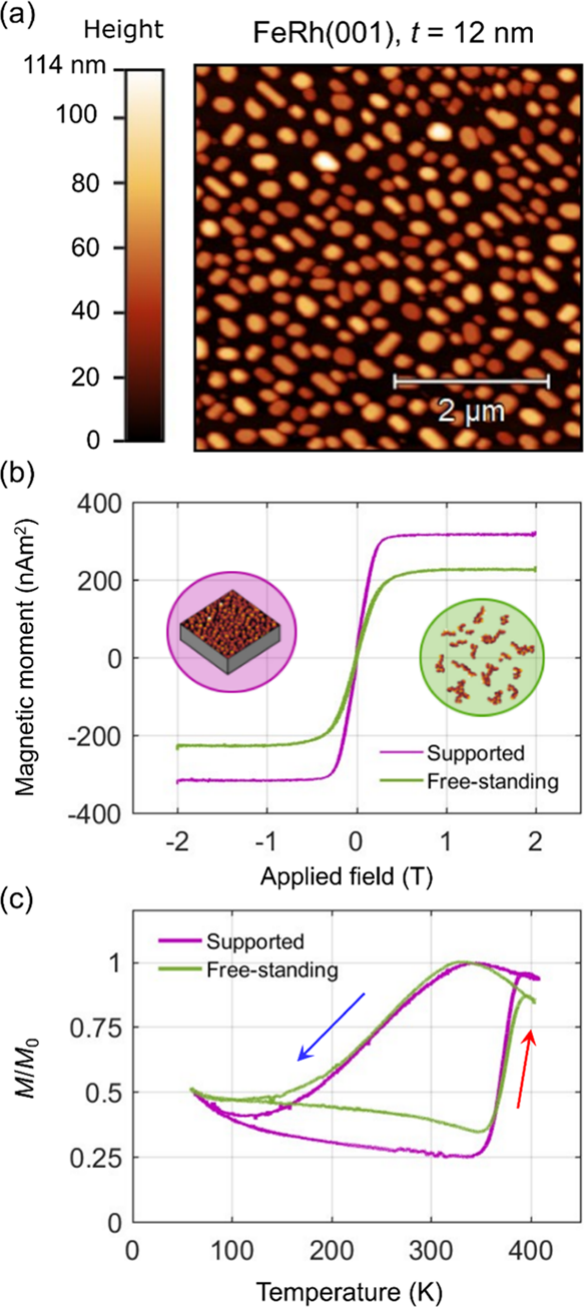
Metamagnetism in free-standing FeRh nanoparticles. (a) AFM image of FeRh(001) islands before etching. (b) Magnetization vs applied field for the supported islands and the released nanoparticles at 400 K. The inset in (b) shows schematics of the supported and free-standing FeRh nanomagnets. (c) Normalized magnetic moment vs temperature at 1 T for the supported and detached FeRh islands. The arrows indicate the heating and cooling cycles.

We released the supported FeRh(001) nanoislands on MgO(001) from the substrate in order to study their magnetic properties as free-standing nanoparticles. Figure 7a shows an AFM image of FeRh nanoislands assembled from a 12 nm-thick film via solid-state dewetting, featuring typical sizes of 200 nm and below. Nanoislands were separated from the substrate via chemical etching of MgO (see Experimental Section).

Figure 7b shows the field-dependent magnetic moment at 400 K for the FeRh nanoislands before and after being released from the substrate. The measured maximum magnetic moment of 0.32 μA m^2^ for the supported islands agrees well with that of a nominally 12 nm-thick film with a magnetization value of 1120 kA m^–1^.^16,17^ Likewise, the maximum magnetic moment value of 0.23 μA m^2^ measured for the released nanoparticles allows estimating that ∼72% of the nanoislands were recovered. Considering the nanoparticles as platelets with an average nanoparticle diameter of 200 and thickness of 60 nm (Figure S11, Supporting Information), it can be estimated that ∼1.2 × 10^8^ nanoparticles were obtained after separation.

The temperature-dependent normalized magnetic moment is shown in Figure 7c for both nanoislands and nanoparticles. The phase transition characteristics are very similar for the FeRh nanomagnets when supported and released from the substrate, with a virtually identical temperature dependence of the magnetic moment for the heating and cooling cycles. As in the case of supported nanoislands, free-standing nanoparticles show a relatively abrupt increase of the moment during heating and a smoother decrease during cooling (Figure 7c), thus the prominent supercooling behavior being kept after release from the substrate. We conclude that the substrate-induced strain in ∼200 nm-sized nanoislands is largely relaxed as a result of the surface-to-volume ratio increasing upon dewetting, opposite to continuous thin films where detachment from the substrate causes large shifts in the phase transition temperature.^55^

We find a slight difference for supported and free-standing FeRh nanomagnets in terms of the residual FM moment fraction in the nominal AF phase (see heating cycle in Figure 7c), where it is about ∼20% higher in the latter case. We explain this in terms of the nanoparticle separation process, where nanoparticles in the FM phase were likely captured more efficiently than those in the AF state. The ∼28% fraction of non-recovered nanoparticles would show a comparatively lower amount of residual magnetic moment, explaining the difference. Another possibility is that nanoparticle accumulation could stabilize the FM phase within these clusters, thus suppressing the AF order within a limited fraction of nanoparticles.

## 3 Conclusions

In conclusion, we have investigated self-organization of metamagnetic FeRh nanoislands using sputter deposition. Two different routes lead to the self-assembly of epitaxial and sub-micron nanomagnet arrays on single-crystal oxide substrates. On the one hand, Volmer–Weber nucleation leads to densely packed nanoislands with predominant faceting along the principal axes of FeRh. On the other hand, the growth of a metastable continuous film and subsequent solid-state dewetting lead to multifaceted nanoislands with sizes down to ∼100 nm. The size and shape of nanoislands assembled via dewetting can be controlled via epitaxy and by tuning the deposited FeRh thickness. While we find that the phase transition is strongly suppressed in sub-micron islands nucleated during Volmer–Weber growth, dewetted FeRh islands show preserved metamagnetism.

Tracking the magnetic properties of a single nanoisland upon temperature cycling reveals large confinement effects such as very pronounced supercooling (>150 K) and the absence of phase separation in sub-500 nm nanoislands. The detailed comparison of the specific crystal faceting and magnetic properties of nanoislands assembled via nucleation and dewetting permits establishing that nanoscale morphology has a strong impact on the phase transition characteristics of nanoscale FeRh systems. We find that nanoislands showing a predominant {100} crystal faceting are strongly FM stabilized, while those showing a prevailing {110} faceting can undergo the phase transition to the AF phase.

Self-assembly of FeRh islands on oxide substrates could be further controlled via templated dewetting by making use of pre-patterned substrates or films,^62^ optimizing aspects such as the nanoisland lateral size distribution or enabling the fabrication of regularly spaced arrays.

Finally, we have also released metamagnetic FeRh nanoislands from the substrate using chemical etching and have studied the phase transition characteristics of self-standing nanoparticles. The magnetic properties of the released FeRh islands do not significantly vary with respect to the supported case and exhibit almost identical phase transition temperatures, supercooling behavior, and residual fractions of magnetic moment. We envision the possibility to fabricate more substantial amounts of functional FeRh nanoparticles via sputter deposition and solid-state dewetting on larger area substrates. Based on nanoisland densities of ∼5–10 μm^–2^ and considering the typical nanoisland height and lateral size values obtained here, the utilization of larger-scale wafers^63^ would enable producing ∼10^10^ to 10^11^ nanoparticles by using, for example, 4 in. (102 mm) wafers, thus reaching milligram mass ranges of metamagnetic FeRh in the form of nanoparticle ensembles.

## 4 Experimental Section

### 4.1 Sample Growth and Self-Assembly

FeRh thin films were sputter-deposited onto single-crystal MgO(001), MgO(011), and Al_2_O_3_(0001) substrates (5 × 5 × 0.5mm^3^ in size) from an equiatomic FeRh target in a high-vacuum chamber with a base pressure of 5 × 10^–8^ mbar. All substrates were preheated to 750 K in high vacuum for 1 h in order to outgas and reconstruct the oxide surface. Unless otherwise indicated, FeRh growth was performed at a substrate temperature of 750 K and an Ar pressure of 2.7 × 10^–3^ mbar. The deposition rate for FeRh was calibrated via X-ray reflectivity for continuous films and determined to be 2 nm min^–1^. To fabricate the FeRh nanoislands, thin films were post-growth annealed in situ and in high vacuum at 1100 K for 80 min to induce self-assembly via solid-state dewetting, as well as to improve the bcc-like structural and chemical ordering. The samples were taken out to air after they were cooled down below 373 K.

### 4.2 In Situ Surface Elemental Analysis

The elemental composition of the sample surface during solid-state dewetting was monitored during the course of post-growth annealing using LEIS in uncapped FeRh thin films that were previously sputter-deposited in a separate chamber. The extreme surface sensitivity of LEIS allows providing straightforward identification of elements in the outermost surface layer, with the measured signal intensities reflecting the surface concentration of the detected elements.^64,65^ We have used a 3 keV He ion beam at a scattering angle of 145° to obtain a spectrum of the sample while steadily ramping up the temperature (9 K min^–1^) from 300 to 1100 K. The atomic mass of the target atoms can be obtained by tracking the kinetic energy of the He projectile and following the rules of elastic binary collisions.^66^

### 4.3 Atomic and Magnetic Force Microscopy

AFM/MFM measurements were realized using a Dimension Icon microscope from Bruker Corporation. The majority of the data were acquired by employing commercial MESP probes with a hard magnetic CoCr coating. Their resonance frequency is about 75 kHz, and the spring constant amounts to 3 N m^–1^. Topography (AFM) was acquired in the PeakForce Tapping non-resonant mode, which responds to short-range interactions. MFM is measured in the second pass (interleave) LiftMode via monitoring the phase shift of the oscillating cantilever driven near its resonant frequency. MFM images are acquired in a constant external magnetic field of ∼0.3 T provided by a permanent magnet. The field is applied in an out-of-plane direction to facilitate visualization of the FM phase. High-resolution AFM images were acquired using Olympus OMCL-AC240TS probes with a nominal tip radius of 7 nm, a cantilever resonance frequency of 70 kHz, and a spring constant of 2 N m^–1^. The finite size of the tip apex leads to minor tip convolution artifacts such as edge rounding; yet, it is still sufficiently small to determine the principal crystalline facets of individual islands without performing tip deconvolution (e.g., available via the Gwyddion software^67^). Temperature control during AFM/MFM measurements is achieved via a custom-made sample holder based on Peltier modules and provides a regulation in the range of 290–380 K at ambient conditions. AFM/MFM data were analyzed and visualized using the open-source Gwyddion software.^67^ The modeling and depiction of individual nanoisland morphologies were realized using the WulffPack Python package,^68^ which enables the prediction of the Wulff and Winterbottom constructions of a given nanocrystal provided its crystallographic structure, facet-dependent surface energies, and the nanocrystal/substrate interfacial formation energy are known. The analysis of experimental nanoisland morphologies and crystallographic facet determination was performed with the assistance of the in-built facet analysis tool in Gwyddion, in combination with modeling in WulffPack.

### 4.4 Structural Analysis

XRD measurements were performed using a Rigaku SmartLab 9 kW diffractometer with Cu K_α_ radiation (λ = 1.5406 Å) using a double-bounce Ge(022) monochromator and a 5° Soller slit in the incident and diffractive optics, respectively.

### 4.5 Electron Microscopy Imaging

SEM images were acquired using a high-resolution Verios 460L microscope by FEI using indistinctively secondary electrons or backscattered electrons.

### 4.6 Magnetization Measurements

Temperature-dependent magnetization measurements were performed via VSM using a Quantum Design VersaLab magnetometer in the temperature range of 55–400 K and under an in-plane applied magnetic field of 1 T. The magnetization of nanoisland samples was calculated assuming an FeRh volume equivalent to a film with the deposited nominal thickness. All data are presented after subtracting the diamagnetic substrate contribution.

### 4.7 Etching and Separation of FeRh Nanoislands from the Substrate

FeRh nanoislands supported on MgO(001) substrates were released in a 0.3 M solution of the disodium salt of ethylenediaminetetraacetic acid (EDTA), which was reported effective to etch MgO substrates (rate ∼0.8 μm h^–1^) and release continuous metallic films.^69^ Ultrasonication did not produce any visible nanoisland detachment,^70^ most likely due to the strong epitaxial clamping to the substrate. The required amount of EDTA disodium salt for a 0.3 M solution was dissolved with the aid of a magnetic stirrer at 363 K to speed up the process. Subsequently, MgO(001) substrates with the fabricated FeRh nanoislands on top were inserted in the solution and kept in an oven at 348 K for ∼30–90 min, until reaching the release of the majority of nanoislands from the substrate. The released FeRh nanoparticles were separated from the EDTA disodium salt solution using a magnetic separation procedure and collected in a polypropylene capsule suitable for VSM measurements (Figure S12, Supporting Information).

## Supporting Information Available

XRD, SEM, and AFM data with the corresponding size distribution analysis of self-assembled FeRh nanoislands; in situ surface elemental analysis during dewetting; impact of density and size of nucleated holes on the resulting size of nanoislands; epitaxial relations for FeRh nanoisland growth on different single-crystal oxide substrates; AFM/MFM and VSM data of nanoislands on different substrates; crystal facet analysis of nanoislands on different substrates; nanoisland size analysis before etching and nanoparticle separation; description of nanoparticle separation process; and a note on Wulff-Kaischev’s theorem for a supported nanocrystal (PDF)
