# Correction to “Preserving Metamagnetism in Self-Assembled FeRh Nanomagnets”

**DOI:** 10.1021/acsami.3c06789

**Published:** 2023-05-25

**Authors:** Lucie Motyčková, Jon Ander Arregi, Michal Staňo, Stanislav Průša, Klára Částková, Vojtěch Uhlíř

In the original version of this article on p. 8657 (https://pubs.acs.org/doi/10.1021/acsami.2c20107), Figure 3e contains the wrong magnetic force microscopy (MFM) data set. The displayed MFM image corresponds to the same sample and same magnetic state as referred to, but has a larger lateral size of 10 × 10 μm^2^. In the revised graphic provided below, the correct MFM image of 5 × 5 μm^2^ is shown in Figure 3e, such that MFM data are in accordance with the same sample area described by the topography data shown above it in Figure 3b. This correction does not alter the conclusions of this work.

**Figure 3:**
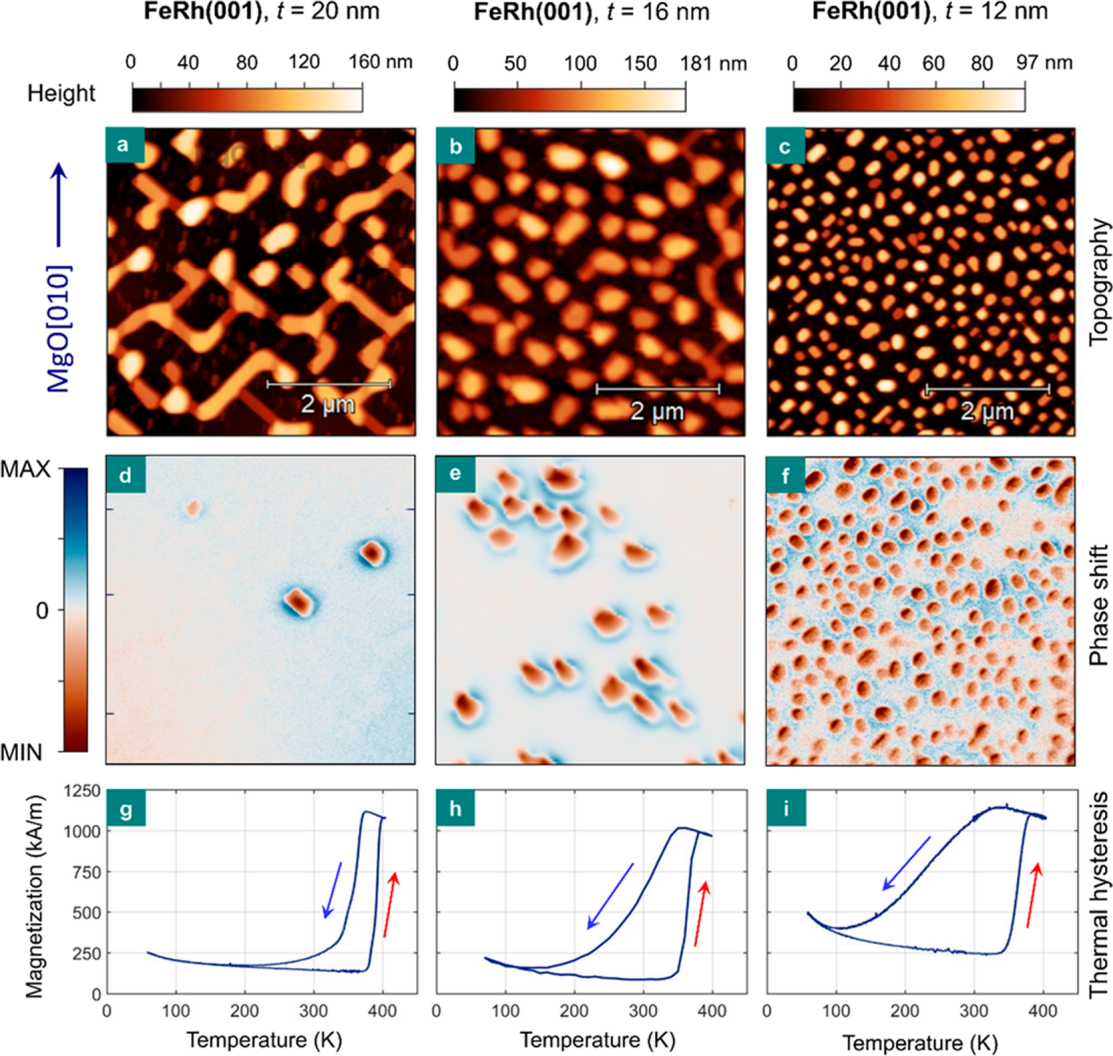
Room-temperature magnetic properties and phase transition in FeRh nanoislands. (a–c) AFM topography images over a 5 × 5 μm^2^ area of nanoisland samples with *t* = 20, 16, and 12 nm. (d–f) Room-temperature MFM measurements over the same sample area. The inset in (a) indicates the crystallographic in-plane direction of the micrograph, which is also valid for all panels (b–f). (g–i) Temperature dependence of the magnetization in the range of 55–400 K for the samples described above. The arrows indicate the heating and cooling cycles in the thermal hysteresis.

