# Supplementary material for: Preserving Metamagnetism in Self-Assembled FeRh Nanomagnets
Source: ACS Appl Mater Interfaces. 2023 Jan 31;15(6):8653–65. doi: 10.1021/acsami.2c20107 (PMC10016751; doi:10.1021/acsami.2c20107)
Supplement: Supplementary file 1 — am2c20107_si_001.pdf [file am2c20107_si_001.pdf]

Supporting Information

Preserving Metamagnetism in Self-Assembled FeRh Nanomagnets

Lucie Motyčková[1], Jon Ander Arregi[1,*], Michal Staňo[1], Stanislav Průša[1,2], Klára Částková[1,3], and Vojtěch Uhlíř[1,2,*]

[1] CEITEC BUT, Brno University of Technology, Purkyňova 123, 612 00 Brno, Czech Republic

[2] Institute of Physical Engineering, Brno University of Technology, Technická 2, 616 69 Brno, Czech Republic

[3] Department of Ceramics and Polymers, Brno University of Technology, Technická 2, Brno 616 69, Czech Republic

*Email: ja.arregi@ceitec.vutbr.cz, vojtech.uhlir@ceitec.vutbr.cz

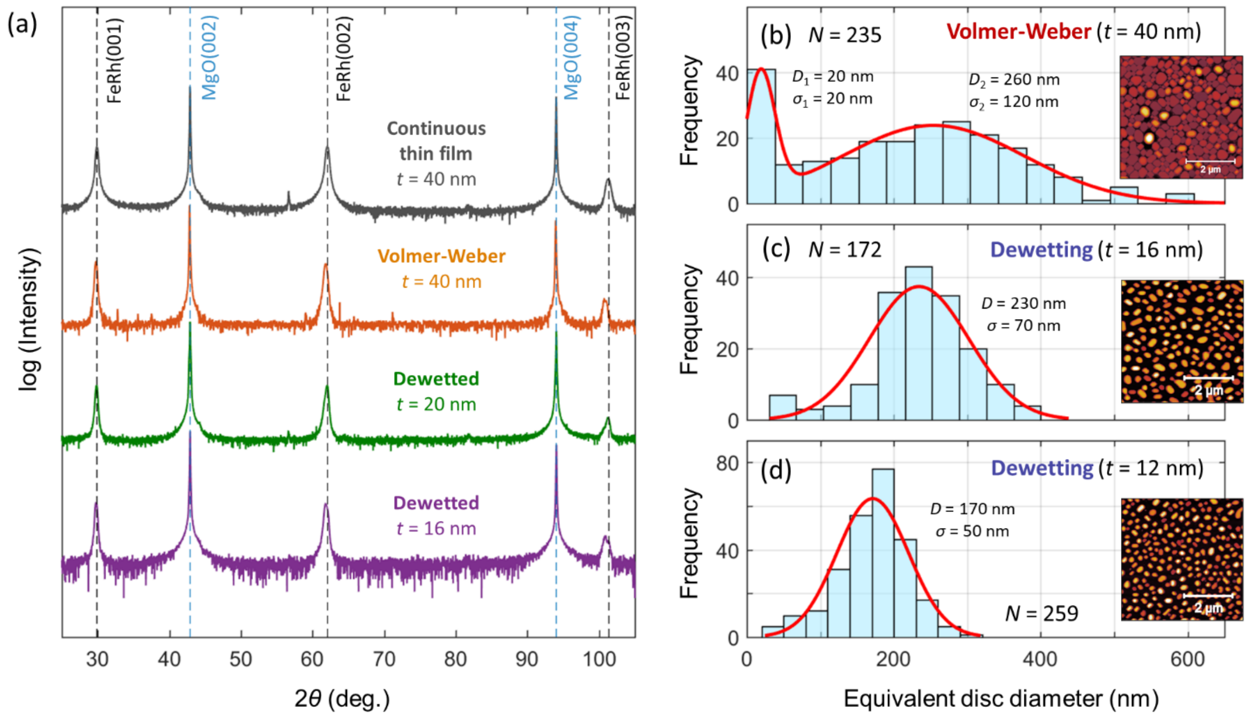

Figure S1. Crystallographic texture and size distribution analysis of self-assembled FeRh nanoislands. (a) XRD θ-2θ symmetric scans for FeRh nanoisland samples assembled via Volmer-Weber nucleation (t = 40 nm) and solid-state dewetting (t = 12, 16 nm) on MgO(001) substrates. All samples show a prominent (001) out-of-plane texture of the CsCl-type structure of FeRh. XRD data for a continuous FeRh film (t = 40 nm) is shown for comparison. (b)-(d) show the size distribution of N nanoislands evaluated from AFM topography data over a 5 × 5 μm^2 area (see insets). Histograms of the equivalent disc diameter are displayed for (b) Volmer-Weber nucleated nanoislands with t = 40 nm, as well as dewetted nanoislands with (c) t = 16 nm and (d) 12 nm. Volmer-Weber nanoislands in (b) show a bimodal distribution function featuring a large presence of small, sub-50 nm islands and a broad distribution of larger ones centered at the 260-nm-diameter size. Dewetted islands in (c), (d) exhibit narrower nanoisland size distributions with central diameter values 230 and 170 nm for t = 16 and 12 nm, respectively. The red solid lines in (b)-(d) represent fits to normal distribution functions, with D being the center diameter and σ the standard deviation.

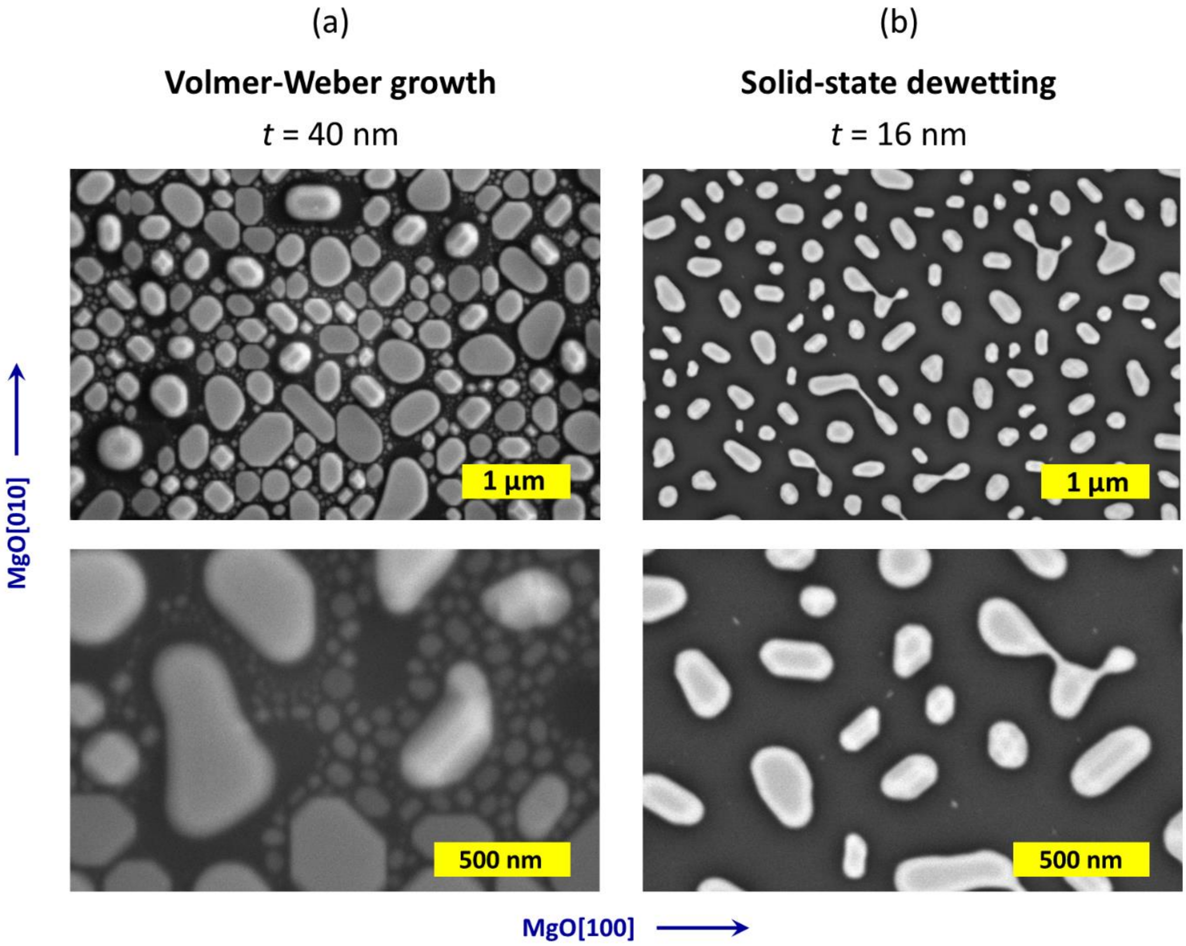

Figure S2. Electron microscopy images of FeRh nanoislands. Top-view SEM micrographs of FeRh nanoislands on MgO(001) formed via (a) Volmer-Weber nucleation (t = 40 nm) and (b) solid-state dewetting (t = 16 nm). The high magnification images in the bottom panel of (a) reveal the ubiquitous presence of sub-50 nm nanoislands intercalated between larger ones (100-400 nm), pointing to a growth mechanism based on island nucleation. Nucleated FeRh islands show predominant faceting for the {100} crystallographic planes. The bottom panel in (b) shows that dewetted nanoislands are well separated with no tiny islands in between. Dewetted islands also show a more diverse crystallographic faceting, giving them a more rounded appearance.

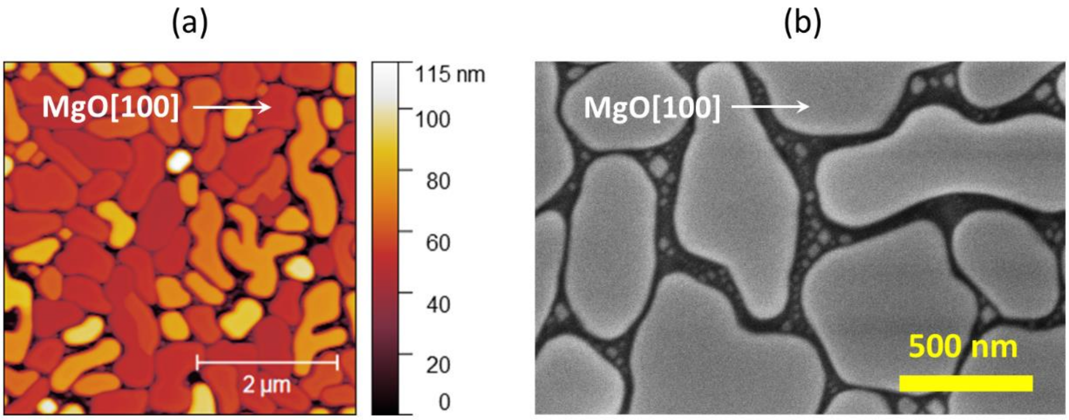

Figure S3. Morphology of high-temperature grown FeRh sample (t = 40 nm). (a) AFM topography and (b) SEM images of an FeRh sample grown at 1100 K and post-growth annealed at the same temperature for 80 min. The sample features islands with sizes in the ~ 0.5-1 µm range and more arbitrary shapes. The SEM image in (b) reveals the existence of intercalated sub-50 nm nanoislands.

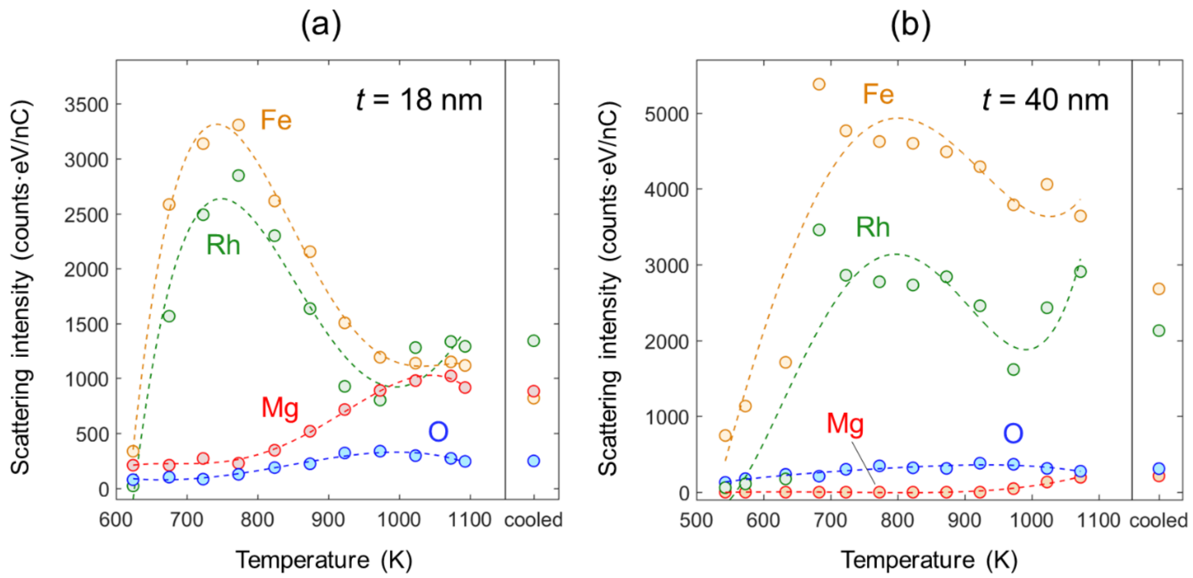

Figure S4. *In-situ* surface elemental analysis during dewetting of FeRh. Temperature-dependent LEIS intensity (integral of the energy-dependent peaks) for atomic Fe, Rh, Mg and O during *in-situ* annealing of FeRh films. Measurements are shown for (a) 18-nm- and (b) 40-nm-thick FeRh films on MgO(001). The dotted lines are a guide-to-the-eye obtained by a cubic spline of the measured data. The scattering intensity of each atomic species (representing their relative sample surface coverage) are shown while steadily ramping up the temperature at a rate of 9 K min^{-1}. The initial increase of the Fe and Rh atomic signals up to 700-800 K is indicative of surface dirt degassing (e.g., hydrocarbons). The subsequent (>800 K) decrease of the Fe and Rh scattering intensity is accompanied by an increase of the Mg and O signals, pointing towards dewetting of the FeRh thin film on the MgO substrate. These signatures are considerably strong for the 18-nm-thick film, while being less pronounced for the thicker 40 nm film, thus confirming the observed thickness dependence of the degree of dewetting in the FeRh/MgO system (Figure 1b of the main manuscript). The ‘cooled’ data points in the right-hand side of the graphs (performed upon cooling the sample down to 523 K right after reaching 1100 K) indicate that the dewetting-induced changes in the LEIS scattering intensity for Fe, Rh, Mg and O are permanent.

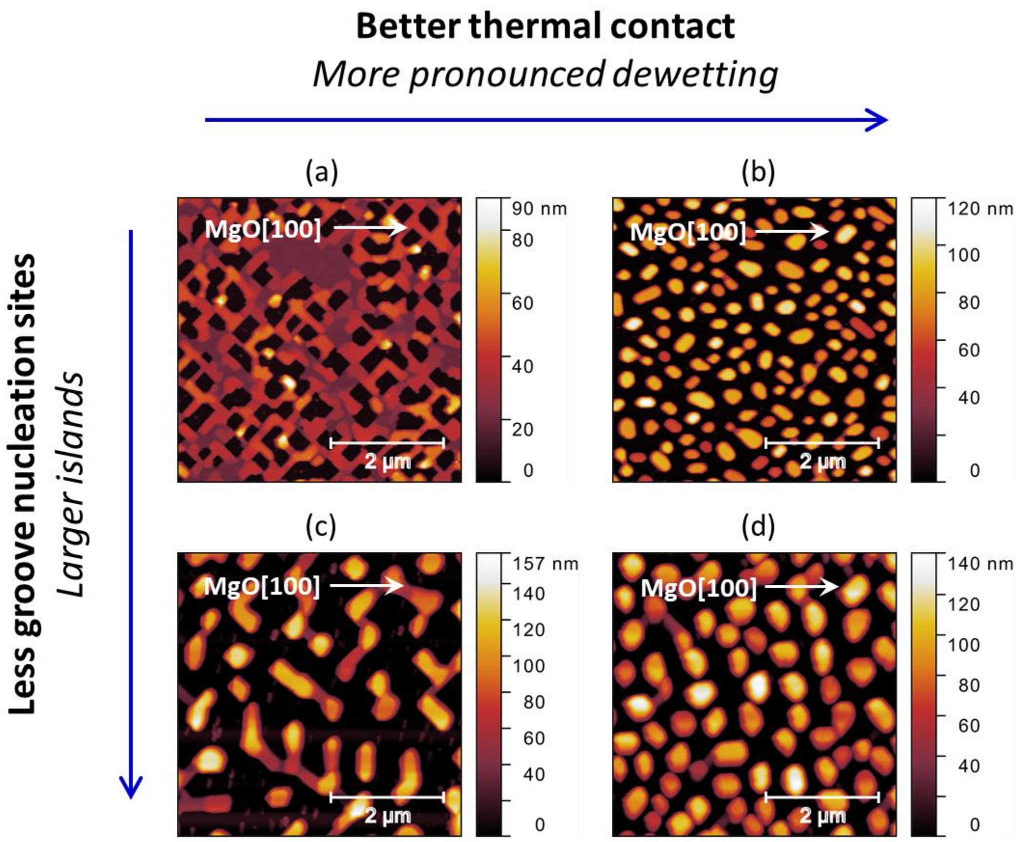

Figure S5. Impact of density and size of nucleated holes on the resulting size of nanoislands formed via solid state-dewetting. AFM topography scans over a 5 × 5 µm^2 area for four different dewetted FeRh(001)/MgO(001) samples with a nominal thickness of t = 16 nm. The samples were fabricated using the same nominal procedures for growth and annealing. We find that the final FeRh nanoisland morphology strongly depends on extrinsic factors such as the substrate-to-sample-holder thermal contact during fabrication (deposition and annealing), or the presence of defects and contamination on the substrate. We identify the general trends that impact the size and density of nanoislands. On the one hand, a better thermal contact of the substrate with the heater element during fabrication will lead to a more advanced dewetting stage and higher fragmentation of the film into nanoislands; compare (a) and (b), or (c) and (d). On the other hand, a larger number of defects and contaminants on the substrate lead to an increased presence of groove nucleation sites in the film, thus resulting in a higher density of void formation and smaller nanoislands; compare (a) and (c), or (b) and (d).

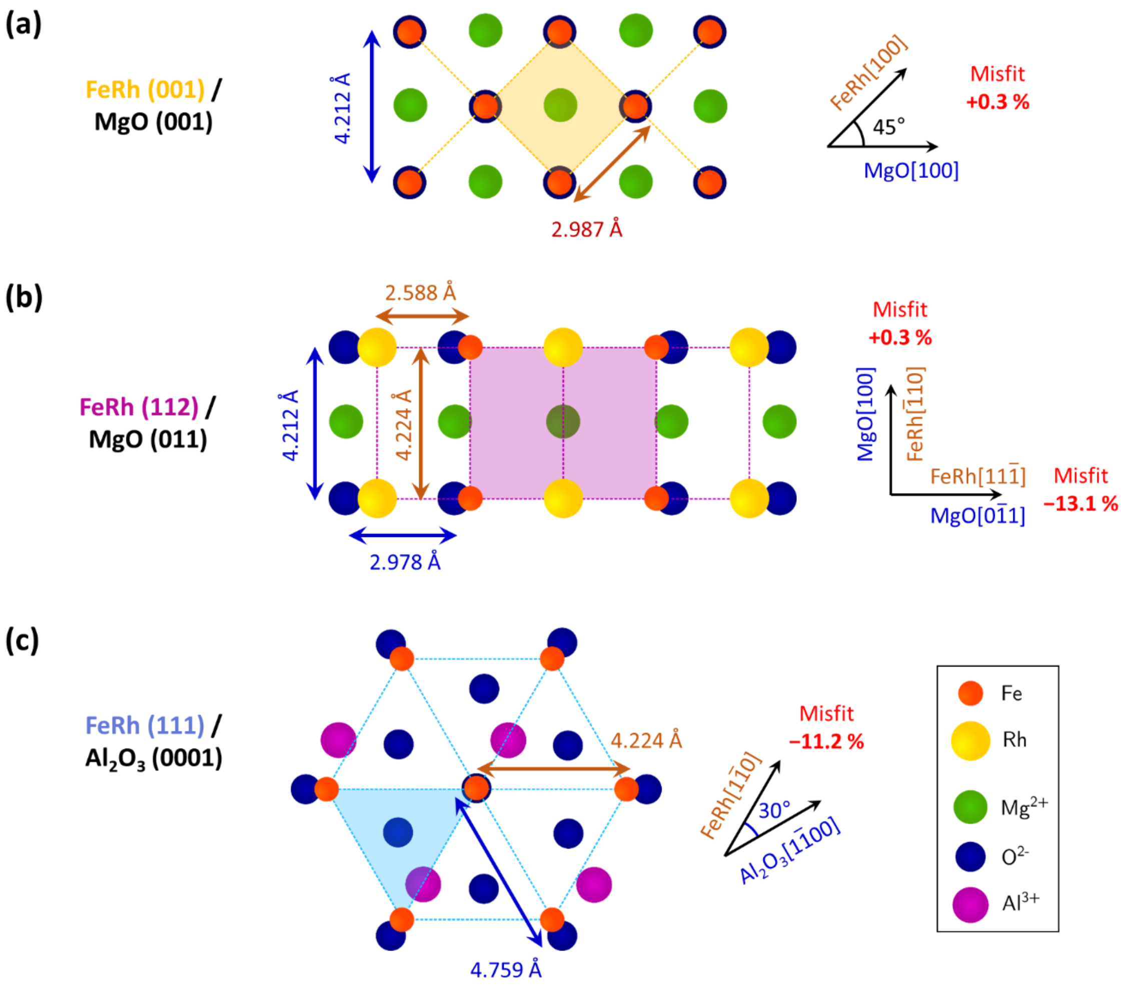

Figure S6. Epitaxial relations for FeRh growth on single crystal oxide substrates. Schematics of in-plane epitaxy for the (a) FeRh(001)/MgO(001), (b) FeRh(112)/MgO(011), and (c) FeRh(111)/Al_2O_3(0001) systems. The characteristic lattice dimensions, orientations, and direction-dependent lattice misfit values are indicated in the graphics. The legend in the bottom-right panel indicates the colors used to represent the different atoms and ions in the lattice.

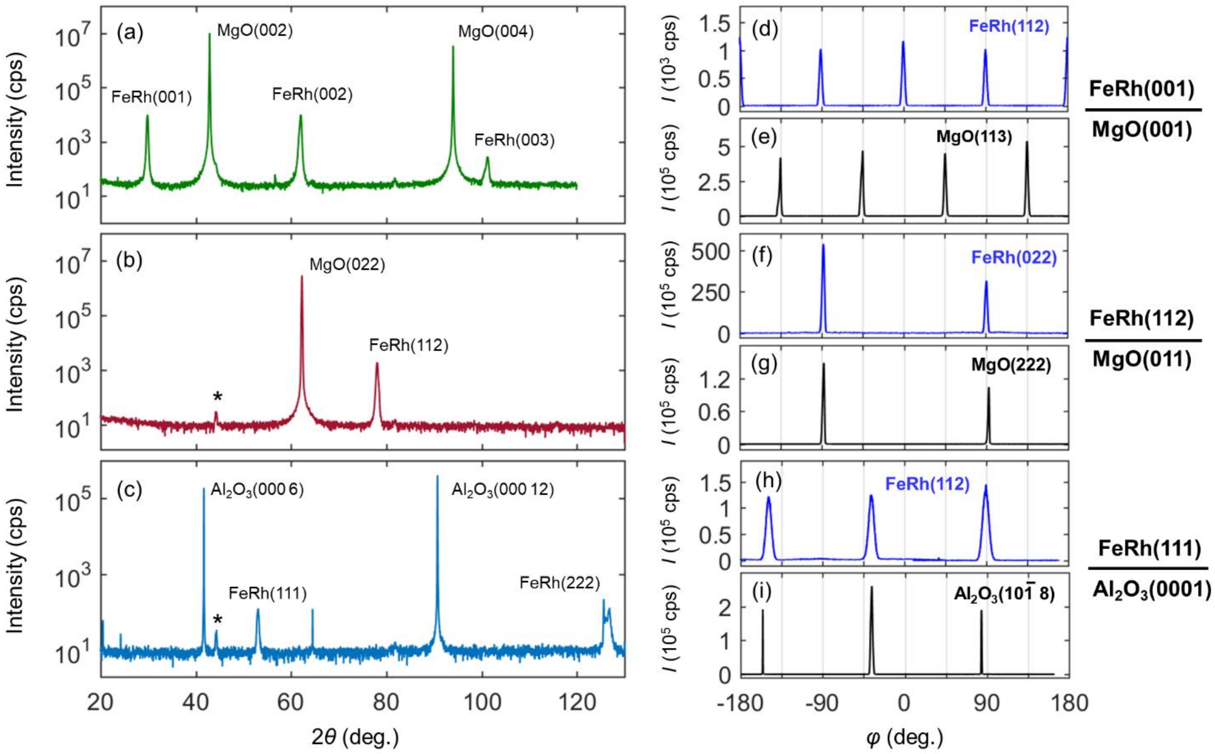

Figure S7. X-ray diffraction of self-assembled FeRh nanoislands (t = 16 nm) on different substrates formed via solid-state dewetting. The left panel exhibits symmetric $\theta/2\theta$ scans showing the out-of-plane crystallographic texture of the epitaxial (a) FeRh(001)/MgO(001), (b) FeRh(112)/MgO(011), and (c) FeRh(111)/Al_2O_3(0001) nanoislands. The peaks labelled as '*' correspond to the sample holder stage. On the right panel, azimuthal φ-scans demonstrating the in-plane epitaxial relationships for the FeRh nanoislands on the three different substrate systems: (d), (e) FeRh(001)[100] || MgO(001)[110]; (f), (g) FeRh(112)[$\underline{1}$10] || MgO(001)[100]; (h), (i) FeRh(111)[11$\underline{2}$] || Al_2O_3(0001)[10$\underline{1}$0].

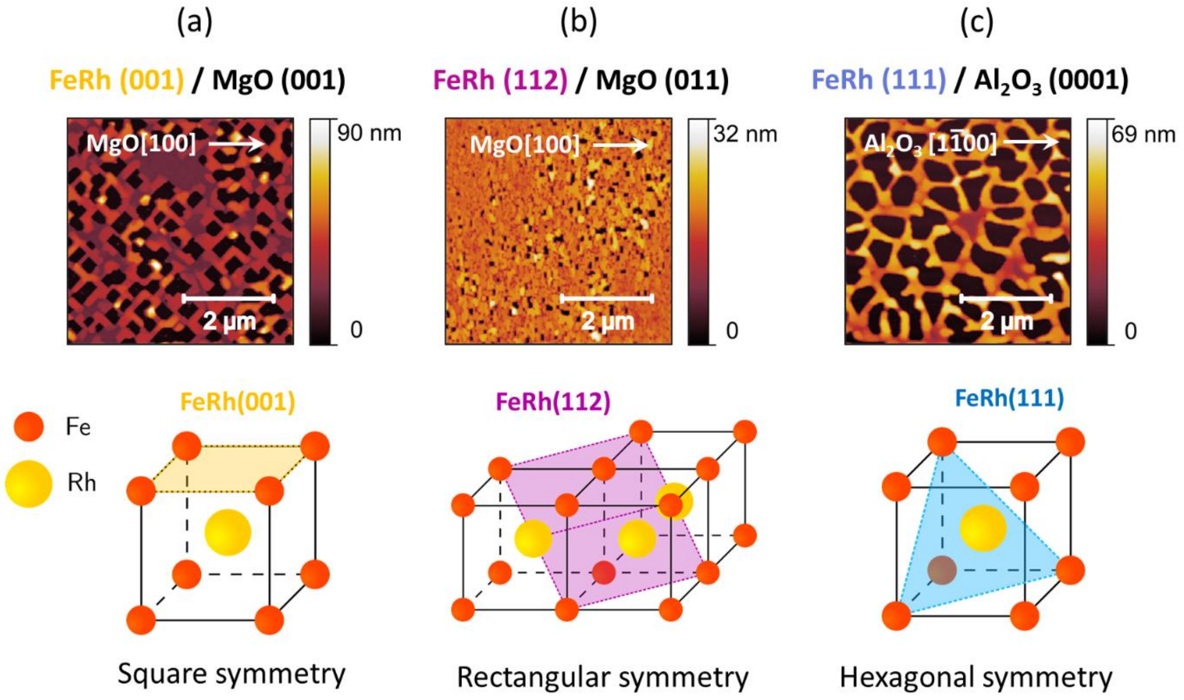

Figure S8. Morphology of partially dewetted epitaxial FeRh films. AFM microscopy images of FeRh films on (a) MgO(001), (b) MgO(011), and (c) Al_2O_3(0001) substrates (with a nominal film thickness of 16, 16, and 12 nm, respectively) representing early stages of solid state dewetting. The samples were obtained upon shortening the annealing time or by the reduced thermal contact of the substrate with the heater stage (e.g., due to the insufficient clamping pressure when mounting the substrate). The morphology of the discontinuous films is characterized by the anisotropic nucleation of holes, these having a specific shape that is given by the crystallographic symmetry of the film.

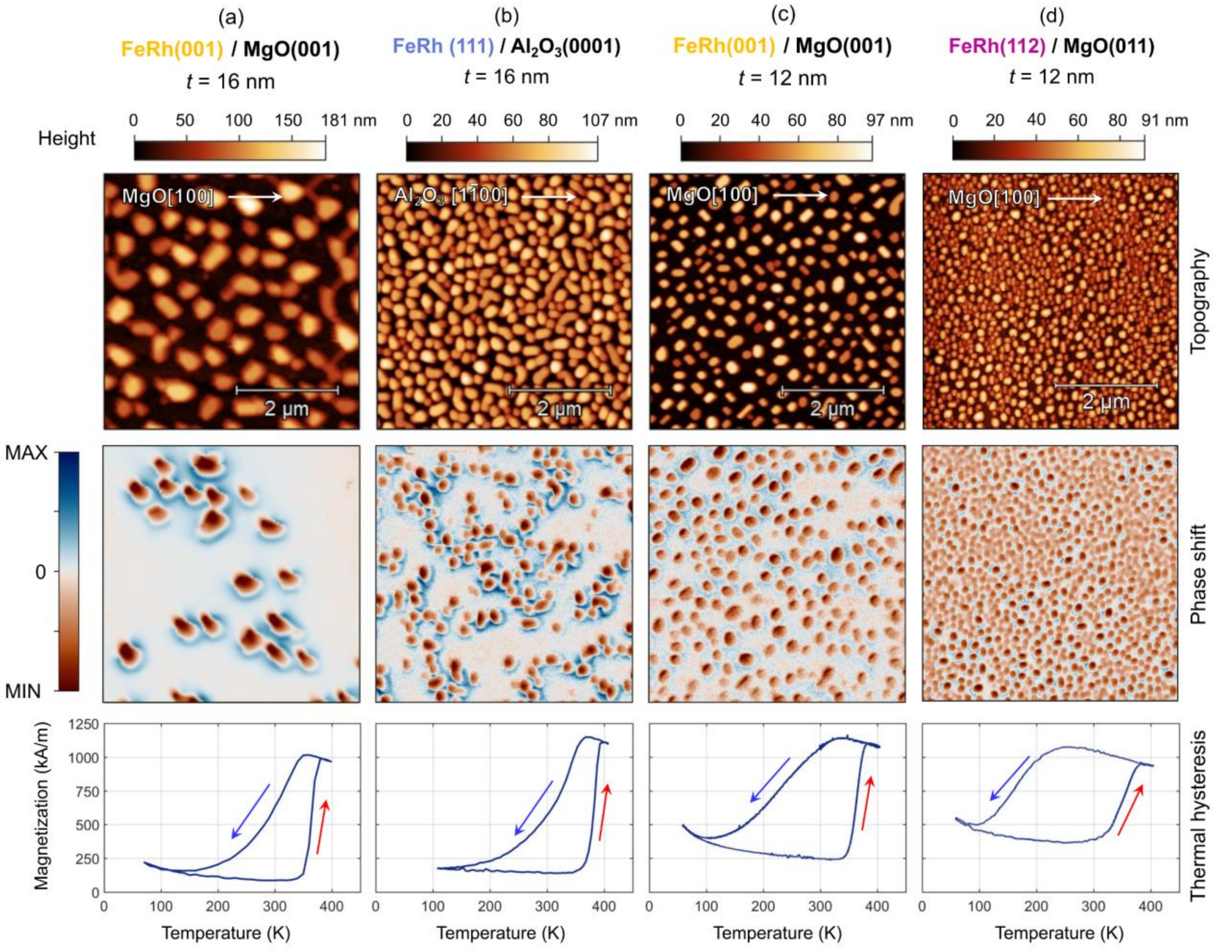

Figure S9. Morphology and magnetic properties of FeRh nanoislands assembled via solid-state dewetting on different substrates. (a) FeRh(001)/MgO(001), *t* = 16 nm; (b) FeRh(111)/Al_2O_3(0001), *t* = 16 nm; (c) FeRh(001)/MgO(001), *t* = 12 nm; (d) FeRh(112)/MgO(011), *t* = 12 nm. AFM topography images and room-temperature MFM measurements are shown over a 5 × 5 μm^2 area in the top and central rows, respectively. The bottom row of panels exhibit temperature-dependent magnetization measurements. Upon comparing FeRh(001) and FeRh(111) nanoislands with *t* = 16 nm, it is seen that the FeRh(111) ones are of smaller characteristics size, as a result of the larger epitaxial mismatch of FeRh with the Al_2O_3(0001) substrate. Their phase transition characteristics are similar, with about a half or less of the nanoislands being FM at room temperature upon cooling from 400 K. In the case of the samples with *t* = 12 nm in (c), (d), the thermal hysteresis is substantially broad during cooling, which is indicative of pronounced supercooling. The majority of islands are FM stabilized at room temperature upon cooling them down from 400 K. FeRh(112) nanoislands exhibit substantially smaller sizes (~100 nm) compared to the FeRh(001) ones, originating from the very large epitaxial mismatch of FeRh on MgO(011).

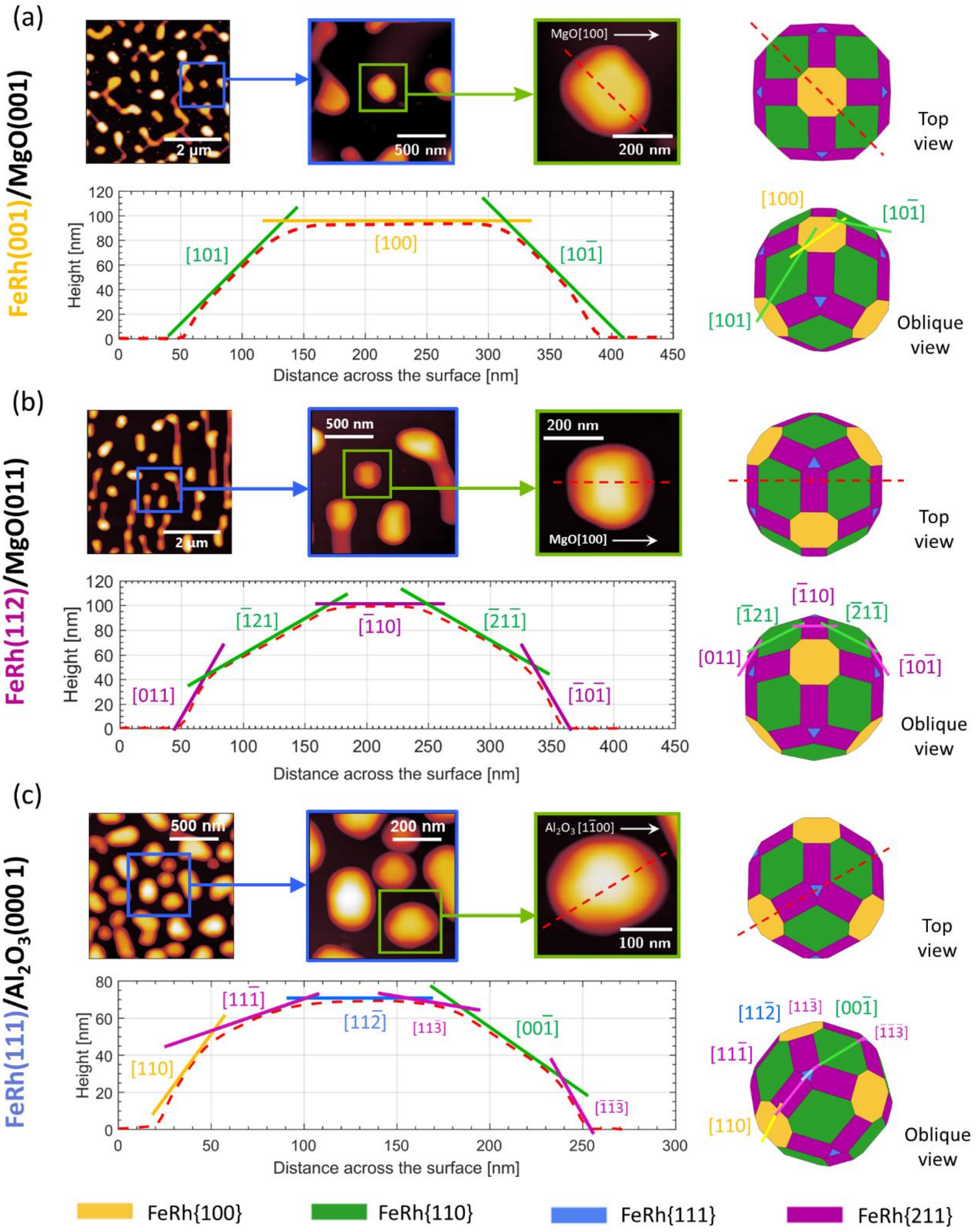

Figure S10. Crystal facet analysis of FeRh nanoislands via topographic line-scan profiles in the (a) FeRh(001)/MgO(001), (b) FeRh(112)/MgO(011), and (c) FeRh(111)(Al_2O_3(0001) systems. AFM topography images (identical datasets as in Figure 2 of the main manuscript) are shown with accompanying line-scan profiles. The crystallographic directions along the line-scan are identified and indicated in the plots. The Wulff constructions including (100), (110), (111) and (211) planes are shown on the right hand side for each case, where the AFM line-scan trajectories are indicated in the top and oblique views. The colored legends at the bottom of the figure indicate the different crystal facet families.

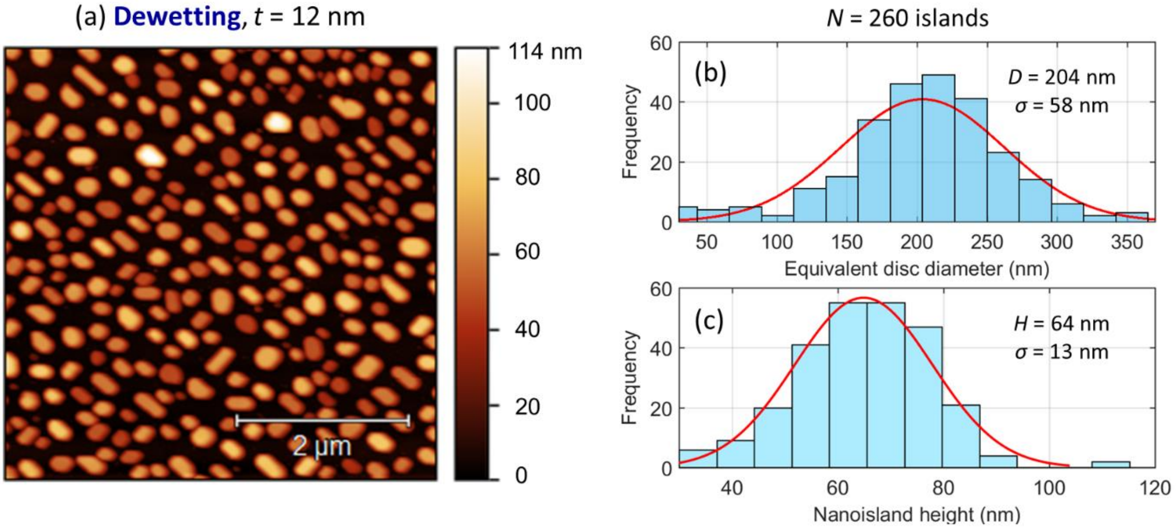

Figure S11. (a) AFM image over a 5×5 μm^2 area of dewetted FeRh nanoislands (t = 12 nm) that were etched away from the MgO(001) substrate. (b), (c) Histograms of the equivalent disc diameter and height for the nanoislands (N = 260) shown in (a). Central diameter and height values of D = 204 nm and H = 64 nm are obtained by fitting the histograms to a normal distribution function.

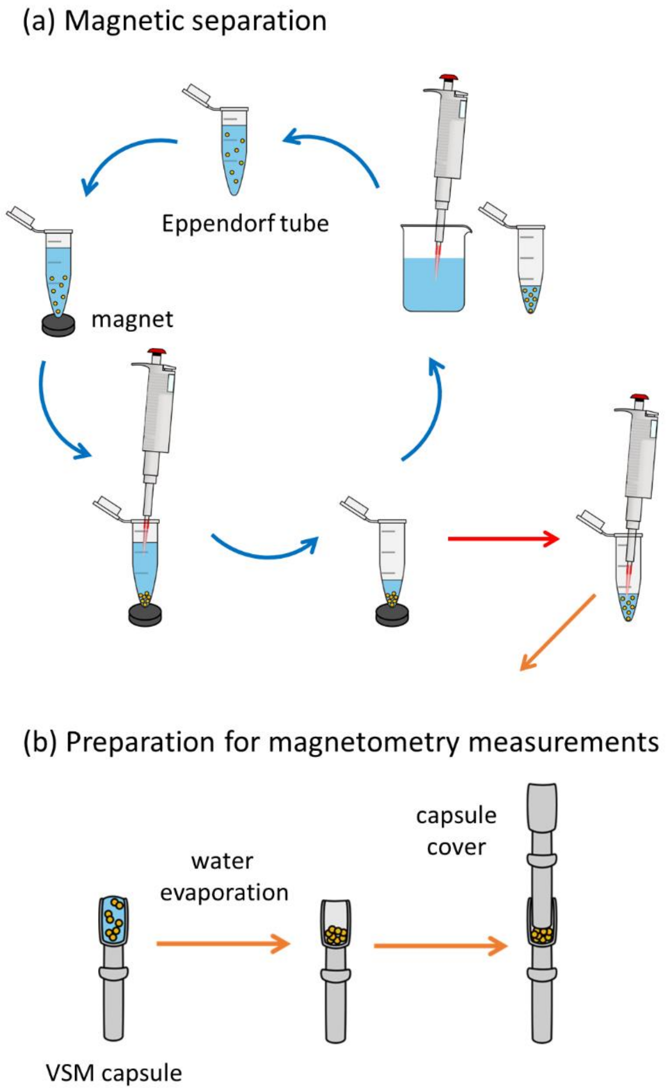

Figure S12. (a) Schematics of nanoparticle separation process. The nanoparticles are initially dispersed or forming clusters in the EDTA disodium salt solution warmed to ~ 348 K within an Eppendorf tube. The solution is briefly heated to 363 K in order to induce the AF-to-FM transition in as many particles as possible. Afterwards, a large Nd-Fe-B permanent magnet providing a field of ~ 0.5 T at about ~ 3 mm from its surface is approached to the bottom part of the tube, causing the nanoparticles in the FM phase to agglomerate in this part. The excess solution from the top is removed using a pipette and the remaining content of the tube is subsequently diluted with deionized water. The process is repeated a few times in order to remove the EDTA disodium salt in the tube. (b) Finally, the magnet is removed, and the reduced liquid volume is captured using a pipette, transferring it to a clean polypropylene capsule for VSM measurements. The water is let evaporate before closing the capsule prior to VSM measurements. The typical fraction of collected nanoparticles constituted about 40-to-70% of the supported nanoislands.

Note S1: Wulff-Kaischev's theorem for a supported crystal

By assuming that the self-assembled FeRh nanoislands are elastically relaxed, the Wulff-Kaischev's theorem predicts the equilibrium shape of a supported crystal on a substrate, mathematically relating the occurrence and geometry of the crystal facets with their surface energies and the formation energy of the interface between the crystal and the substrate. The construction can be concluded considering that the following ratio remains constant for the different facets i of the crystal

$$\frac{\gamma_{\mathrm{i}}}{h_{\mathrm{i}}} = \frac{\gamma_{\mathrm{S}} - \gamma_{\mathrm{int}}}{h_{\mathrm{int}}} \tag{S1}$$

where h_{i} is the distance from the nanocrystal's geometric center to a crystallographic facet i, γ_{i} is the surface energy of the facet, γ_{S} is the surface energy of the substrate, and γ_{int} is the interface formation energy (see Figure S14). In addition, h_{int} is the truncation height of the nanocrystal from its geometrical center. Here, the case $h_{\mathrm{int}} > 0$ is considered, which leads to a supported nanocrystal truncated from above its center (see Figure S14).

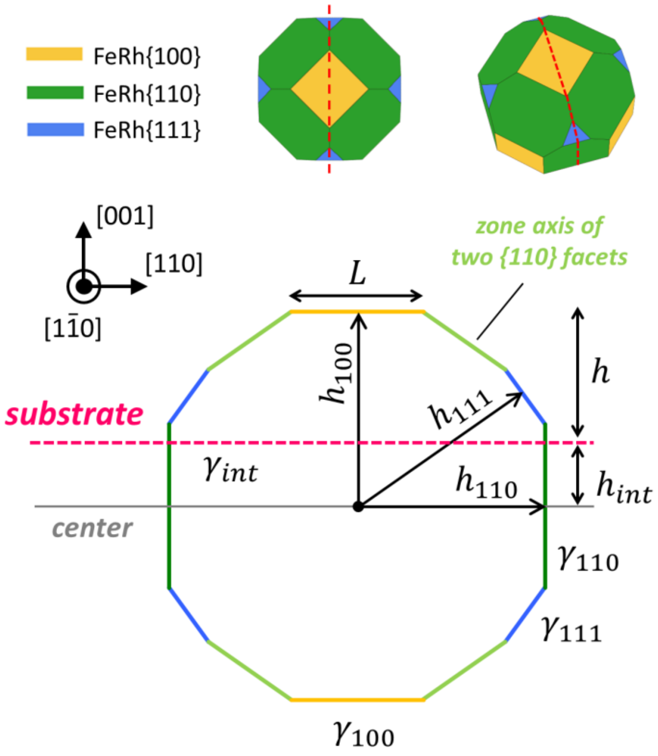

Figure S13. Depiction of the Winterbottom construction. On the top, schematics of a (001) oriented FeRh nanoisland truncated from above its center. The red dashed lines indicate the cutting plane ([1$\bar{1}$0]) along which the nanoisland morphological attributes were measured. On the bottom, definition of the nanoisland attributes used for the Wulff-Kaischev construction.

Considering {100}, {110} and {111} planes, Equation S1 can be rewritten as

$$\frac{\gamma_{100}}{h_{100}} = \frac{\gamma_{110}}{h_{110}} = \frac{\gamma_{111}}{h_{111}} = \cdots = \frac{\gamma_{S} - \gamma_{int}}{h_{int}}. \tag{S2}$$

For the FeRh nanoislands investigated here, $h_{int} > 0$ and thus $\gamma_{int} > \gamma_{S} \approx 1.17$ J m^{-2}. Setting $h_{int} = h_{100} - h$, with h being the height of the nanoisland (see Figure S14), leads to

$$h = \frac{\gamma_{100} - \gamma_{S} + \gamma_{int}}{\gamma_{100}} h_{100}. \tag{S3}$$

For $\gamma_{int} < \gamma_{S}$, we have that $h < h_{100}$ (nanocrystal truncated from above).

Furthermore, we can also express the extent of the nanoisland cusp along the [110] direction (see Figure S14) as $L = 4h_{110} - 2\sqrt{2}h_{100}$, a relation that is independent of the nanoisland truncation height h_{int} and is obtained from evaluating the geometry of Wulff construction. Using Equation S2, L can be rewritten as

$$L = \frac{4\gamma_{110} - 2\sqrt{2}\gamma_{100}}{\gamma_{100}} h_{100}, \tag{S4}$$

and the combination of Equation S3 and S4 leads to

$$\frac{h}{L} = \frac{\gamma_{100} - \gamma_{S} + \gamma_{int}}{4\gamma_{110} - 2\sqrt{2}\gamma_{100}} \tag{S5}$$

which relates the measured h/L of the nanoislands to the surface energies of the {100} and {110} facets of FeRh, the surface energy of the substrate γ_{S}, as well as the FeRh/MgO interface formation energy γ_{int}. Equation S5 can be rearranged to obtain

$$\gamma_{int} = (\gamma_{S} - \gamma_{100}) + \left(4\gamma_{110} - 2\sqrt{2}\gamma_{100}\right) \frac{h}{L}, \tag{S6}$$

which is the same expression as in Equation 1 of the main manuscript. The γ_{int} vs h/L expression above can be interpreted as a straight line with a negative intercept ($\gamma_{S} < \gamma_{100}$) and a positive slope.
